# Phosphates Transfer in Pristine and Modified CJMA-2 Membrane during Electrodialysis Processing of Na_x_H_(3−x)_PO_4_ Solutions with pH from 4.5 to 9.9

**DOI:** 10.3390/membranes13070647

**Published:** 2023-07-05

**Authors:** Natalia Pismenskaya, Olesya Rybalkina, Ksenia Solonchenko, Dmitrii Butylskii, Victor Nikonenko

**Affiliations:** Russian Federation, Kuban State University, 149, Stavropolskaya Str., 350040 Krasnodar, Russia; n_pismen@mail.ru (N.P.); olesia93rus@mail.ru (O.R.); sol.ksenia17@yandex.ru (K.S.); dmitrybutylsky@mail.ru (D.B.)

**Keywords:** phosphates transfer, anion exchange membrane, weakly basic fixed groups, bound species, boundary junction, current–voltage curve, proton generation, electrodialysis, current efficiency, energy consumption

## Abstract

Phosphate recovery from different second streams using electrodialysis (ED) is a promising step to a nutrients circular economy. However, the relatively low ED performance hinders the widespread adoption of this environmentally sound method. The formation of “bonded species” between phosphates and the weakly basic fixed groups (primary and secondary amines) of the anion exchange membrane can be the cause of decrease in current efficiency and increase in energy consumption. ED processing of Na_x_H_(3−x)_PO_4_ alkaline solutions and the use of intense current modes promote the formation of a bipolar junction from negatively charged bound species and positively charged fixed groups. This phenomenon causes a change in the shape of current–voltage curves, increase in resistance, and an enhancement in proton generation during long-term operation of anion-exchange membrane with weakly basic fixed groups. Shielding of primary and secondary amines with a modifier containing quaternary ammonium bases significantly improves ED performance in the recovery of phosphates from Na_x_H_(3−x)_PO_4_ solution with pH 4.5. Indeed, in the limiting and underlimiting current modes, 40% of phosphates are recovered 1.3 times faster, and energy consumption is reduced by 1.9 times in the case of the modified membrane compared to the pristine one. Studies were performed using a new commercial anion exchange membrane CJMA-2.

## 1. Introduction

**Figure 1:**
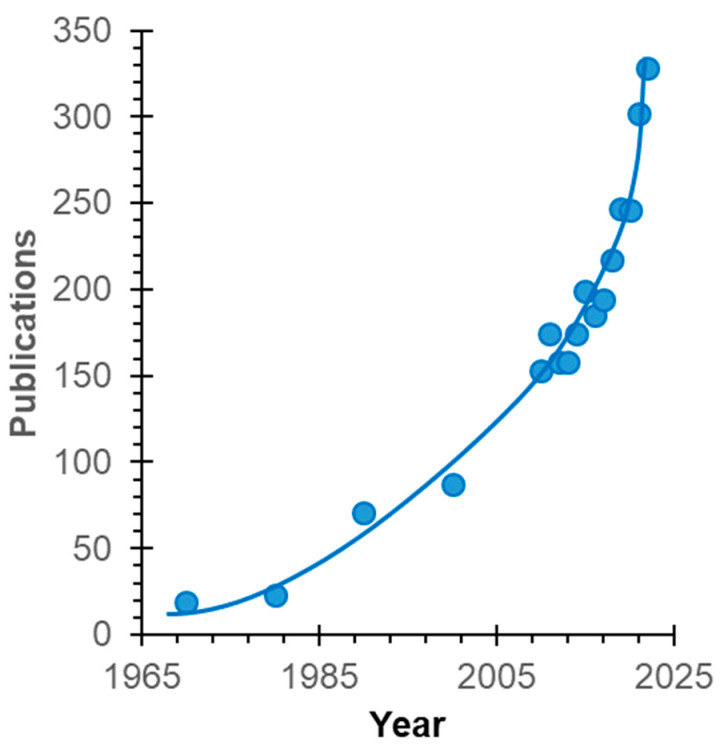
Evolution of the number of publications in Scopus for the keywords “ion AND exchange AND membrane AND phosphates”.

The trend of recent years is the increasing use of ion-exchange membranes for the recovery, separation, and concentration of anions of polybasic organic and inorganic acids. Membrane bioreactors and modules for dialysis, electrodialysis (ED), and capacitive deionization contain these membranes. Already, these membrane technologies are promising for the processing of fermentation broths or waste fermentation effluent [1,2,3,4], agricultural, industrial streams and natural waters [5,6,7,8,9], the selective separation of various acids [10]; tartrate stabilization of wine; demineralization of milk whey; reagent-free correction of the pH of juices and wines [11], or conversion of salts to polybasic acids and vice versa [12,13]. Citrates [2,11,14,15], malates [10,14], tartrates [13], oxalates [7], chromates [16,17], vanadates [18], and sulfates [19] are the most common objects of the application of processes in which ion-exchange membranes are involved. Phosphates are of particular interest, which is accompanied by an avalanche-like increase in scientific publications in recent years (Figure 1).

This interest is caused by the vital need of mankind to organize a circular economy for phosphates [20]. Indeed, on the one hand, they are nutrients and are part of food, biologically active substances, pharmaceuticals, detergents, and fertilizers. Therefore, the consumption of these substances is steadily growing, while the natural reserves of phosphates are decreasing [6]. On the other hand, getting into waste, phosphates cause eutrophication of water bodies and disruption of the ecological balance of the environment [21]. Therefore, the recovery of phosphates from liquid wastes using low-reagent membrane technologies and the return of these substances to production can reduce the anthropogenic and technogenic pressure on the environment and at the same time become a source of valuable raw materials.

Many of the researchers involved in the applied aspects of membrane technologies, in particular, electrodialysis, report relatively low current efficiency and high energy consumption during the processing of streams containing species of polybasic acids [22,23]. Some of them [14,24,25] believe that steric hindrance is one of the main obstacles to the transport of multiply charged anions in conventional anion-exchange membranes (AEMs). Therefore, new generation AEMs were actively developed by many research teams [15,17,26,27,28,29,30] in recent years. Typically, these membranes have an ion exchange matrix that is less rigidly cross-linked compared to conventional membranes [17,26,27]. However, the use of these innovative membranes does not allow for a radical improvement in ED performance, because anions of polybasic acids participate in proton transfer reactions with water and with each other. This feature distinguishes polybasic acid anion transport in AEM systems from the well-studied transport of Cl^−^ and similar anions. For example, the implementation of the “acid dissociation” mechanism causes an increase in the electric charge of the polybasic acid anion (proton-containing phosphate) after its entry into AEM [11,31]. In addition, the generation of protons takes place in both underlimiting and overlimiting current modes. In the case of electrolytes that do not participate in proton transfer reactions, the generation of H^+^, OH^−^ ions, which negatively affects ED performance, is observed only in overlimiting current modes. The coupling of mass transfer with protonation–deprotonation reactions leads to the appearance of two plateaus on the current–voltage curves [14,31,32] and chronopotentiograms [14,33,34] or two Gerischer sub-arches on the spectra of electrochemical impedance [32,35,36]. This generation is carried out with the participation of fixed groups of membranes by the mechanism of water splitting.

Note that the “acid dissociation” mechanism does not explain the significant decrease in the conductivity of AEM [37], the degradation of their characteristics during ED of phosphate-containing solutions [19]. In addition, knowledge of this mechanism does not give an idea of the reason for the increase in the irreversible sorption of anions of polybasic carboxylic acids with an increase in the number of –COOH groups in acid residues and its transformation into the anion –COO– [14,25,38]. Chandra et al. [14,25,38] suggested that the high sorption is caused by the formation of hydrogen bonds between carboxyl groups of the acids and quaternary (–N^+^R_4_) fixed ammonium groups of AEMs.

We assume that these phenomena, as well as the significant energy consumption during ED, can be caused by specific interactions anions of polybasic acids with proton containing weakly basic fixed groups. Most AEMs contain these groups (primary, secondary, and tertiary amines) [26,27]. They are introduced during the synthesis of membranes or are formed during AEMs storage and operation.

It is known that specific interactions (for example, between primary amines and phosphates) cause self-organization of native structures [39,40]. In addition, they provide the formation of dense films with the layer-by-layer method using, for example, poly(styrene sulfonate) and poly(allylamine hydrochloride) [41,42]. Specific interactions with amines underlie the functioning of analytical sensors for determining the concentration of oxygen-containing anions [43,44]. The cause of specific interactions is the implementation of three mechanisms at once [41,43,45,46]: (1) participation of anions of polybasic acids and weakly basic amines in proton transfer reactions; (2) electrostatic interactions; and (3) formation of hydrogen bonds between an anion oxygen and a fixed group hydrogen.

Our study focuses on verification of the hypothesis about the effect of the weakly basic fixed amino groups on the electrochemical characteristics of anion-exchange membranes and ED performance due to their specific interactions with phosphates of the feed solutions. In addition, we are going to test a way to improve ED performance in the processing of phosphate-containing solutions by shielding weakly basic fixed groups with quaternary ammonium bases.

## 2. Materials and Methods

### 2.1. Membranes and Solutions

Studies were performed using an innovative homogeneous anion exchange membrane CJMA-2 (Hefei Chemjoy Polymer Materials Co. Ltd., Hefei, China). This membrane appeared on the market relatively recently. Therefore, knowledge about its properties is fragmentary.

The CJMA-2m membrane was obtained by soaking the pristine (CJMA-2) membranes for 8 h in a 2.5% polyquaternium-22 (CAS No. 53694-17-0, Career Henan Chemical, Zhengzhou, China) aqueous solution. Polyquaternium-22 (PQ-22) is a copolymer of diallyldimethylammonium chloride (DADMAC) and acrylic acid. The PQ-22 polymer contains quaternary amines bonded bidentatically to an aliphatic matrix as well as carboxyl groups in a ratio of 2:1 [47]. PQ-22 concentration in aqueous solution is optimized in previous studies [48].

Heterogeneous cation exchange (MC-40) and anion exchange (MA-41) membranes were auxiliary. Shchekinoazot Ltd. (Shchekinoazot, Pervomaisky, Russia) is their manufacturer. MC-40 and MA-40 characteristics are detailed in [49], as well as in Supplementary Materials (SM).

The experiments were preceded by equilibrating the membranes with 0.02 M NaCl or Na_x_H_(3−x)_PO_4_ solutions. Distilled water (conductivity of 1.0 ± 0.1 μS cm^−1^, pH 5.6 ± 0.1) and analytical grade crystalline salts (OJSC Vekton, St. Petersburg, Russia) of NaCl and NaH_2_PO_4_ were used for preparation of the solutions. The 0.10 ± 0.01 M NaOH solution (Vekton, Russia) was used to adjust the pH of Na_x_H_(3−x)_PO_4_ solutions to 4.5 ± 0.1, 6.6 ± 0.1 and 9.9 ±0.2.

Table 1 summarizes the composition of the studied Na_x_H_(3−x)_PO_4_ solutions. This composition was calculated using the equilibrium dissociation constants of phosphoric acid for the 1st, 2nd, and 3rd stages (see Supplementary Materials).

### 2.2. Methods

Standard characterization of the studied membranes was carried out using optical microscopy, FTIR, determination of contact angles, ion-exchange capacity, water content, and conductivity measurements. A detailed description of the pretreatment of membranes before experiments, as well as the techniques for performing measurements, are given in Refs. [50,51] and in Supplementary Materials.

**Figure 2:**
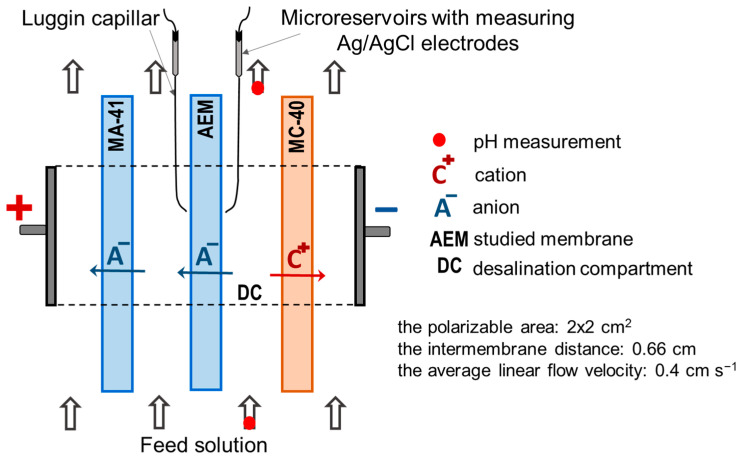
The scheme of the laboratory-scale electrodialysis cell.

Electrochemical characterization of the membranes (voltammetry with simultaneous registration of the desalted solution pH, electrochemical impedance spectroscopy) and determination of ED performance were carried out using a setup and a technique that is repeatedly described, for example, in [31]. Their detailed explanation is presented in Supplementary Materials. A scheme of a laboratory scale electrodialysis cell is shown in Figure 2.

A fresh sample of the membrane was used for each experiment.

Electrodialysis processing of Na_x_H_(3−x)_PO_4_ feed solution with pH 4.5 ± 0.1 and initial concentration 0.03 M was carried out in batch mode. The volume of the solution in the desalination stream was 100 mL. A 0.02 M feed solution with a volume of 1000 mL was circulated through the remaining compartments of the cell. The constant pH value of the feed solution was maintained by adding 0.100 ± 0.001 M NaOH solution to the intermediate tank of the desalination stream.

The temperature was 25.0 ± 0.2 °C in all experiments.

### 2.3. Calculations

In the case of NaCl solutions, as well as Na_x_H_(3−x)_PO_4_ with pH 4.5 or 9.9, which contained predominantly singly charged counterions or doubly charged counterions (Table 1), the limiting current *i_lim_^Lev^* and the thickness of the diffusion boundary layer *δ^Lev^* were calculated using the Leveque equation. The equation was obtained within the framework of the convective diffusion model [52]:

(1) $$i_{\lim}^{Lev}=\frac{z_{1}FDc_{1}^{0}}{h(T_{1}-t_{1})}\left[1.47{\left(\frac{h^{2}V_{0}}{LD}\right)}^{1/3}\right]$$

(2) $$\delta^{Lev}=0.68h{\left(\frac{LD}{h^{2}V_{0}}\right)}^{1/3}$$

The electric charge and electromigration transport number of counterion in the depleted solution at infinite dilution are denoted as *z*_1_ and *t*_1_. The Faraday constant, the diffusion coefficient of electrolyte, and the molar concentration of counterion in the bulk of the feed solution are denoted as *F*, *D*, and $c_{1}^{0}$. The intermembrane distance is the length of the desalination and the average linear solution flow velocities are *h*, *L*, and *V*_0_, correspondently. *T*_1_ is the counterion transport number in the membrane. It was considered equal to 1.

The Na_x_H_(3−x)_PO_4_ solution with pH 6.6 contains about 60% singly charged and 40% doubly charged counterions (Table 1). In this case, the limiting current density was calculated using the equation for the ternary electrolyte [53].

(3) $$i_{\lim}^{Lev}=\frac{F}{\delta }\sum_{k=1}^{2}\left(1-\frac{z_{k}}{z_{A}}\right)D_{k}z_{k}c_{k}^{0}$$

The diffusion coefficient, charge, and molar concentration of counterion *k*, respectively (*k* = 1, 2), are denoted as *D_k_*, *z_k_*, and $c_{k}^{0}$, correspondently. The charge number of the coion is denoted as *z*_A_. An equation similar to Equation (2) is used to determine *δ^Lim^.* The diffusion coefficient of ternary electrolyte, *D_ter_*, was calculated as:

(4) $$D_{ter}=\left[\left(1+\left|\frac{z_{1}}{z_{A}}\right|\right)D_{1}N_{1}+\left(1+\left|\frac{z_{2}}{z_{A}}\right|\right)D_{2}N_{2}\right]\cdot t_{A}$$

where $N_{i}=z_{i}c_{i}^{0}/z_{A}c_{A}^{0}$ is the equivalent fraction of counterion *i* in the bulk solution.

The calculated limiting current densities for 0.02 M Na_x_H_(3−x)_PO_4_ solutions with pH 4.5, 6.9, and 9.9 are equal to 1.64, 2.62, and 3.94 mA/cm^2^, correspondently. In the case of a 0.03 M Na_x_H_(3−x)_PO_4_ solution with pH 4.5, *i_lim_^Lev^* = 2.46 mA/cm^2^.

Knowing the frequency at the peak point of the Gerischer arc, *f_G_*, makes it possible to estimate the effective rate constant of the proton generation reaction, χ [54]:

(5) $$\chi =\frac{2\pi f_{G}}{\sqrt{3}}$$

The following parameters were calculated to determine the ED performance.

The degree of the feed solution desalination, $\gamma_{D}{}^{{}_{}}$, and the degree of P^V^ recovery from the feed solution, $\gamma_{P}{}^{{}_{}}$, were found as:

(6) $$\gamma_{D}=\frac{\kappa^{0}-\kappa^{t}}{\kappa^{0}}$$

(7) $$\gamma_{P}=\frac{c_{P}^{0}-c_{P}^{t}}{c_{P}^{0}}$$

where the indices “0” and “t” correspond to the initial and given moment of the electrodialysis.

The number of protons coming from the anion exchange membrane into the desalination stream during electrodialysis is calculated as:

(8) $$q^{H+}=\frac{c_{T}^{}V_{T}^{}}{S}$$

where $c_{T}{}^{{}_{}}$ and $V_{T}{}^{{}_{}}$ are the concentration and volume of the added alkali (titrant) to maintain pH of the desalination stream, S is the polarizable area of the membrane.

The current efficiency for pentavalent phosphorus contained in phosphates was found by the equation:

(9) $$\eta =\frac{z_{i}^{}F\bar{V^{t}}(c_{p}^{0}-c_{p}^{t})}{n\int_{0}^{t}I(t)dt}$$

Here, *n* is the number of desalination compartments, $\bar{V^{t}}$ is the volume of the solution in the desalination stream after time *t* from the start of the experiment, and *I* is the applied current density (is a constant in the experiments).

The energy consumption, *W*, spent on the desalination of the solution is determined as:

(10) W=∫I(t)Δφ(t)dt

where Δ*φ(t)* was measured using Luggin capillaries, Ag/AgCl electrodes, and the Autolab PGSTAT100 electrochemical complex. In accordance with Equation (10) the integration is carried out from the initial moment to the duration of electrodialysis required to desalt the feed solution by 40%.

## 3. Results

### 3.1. Membranes Characterization

#### 3.1.1. IR Spectra

**Figure 3:**
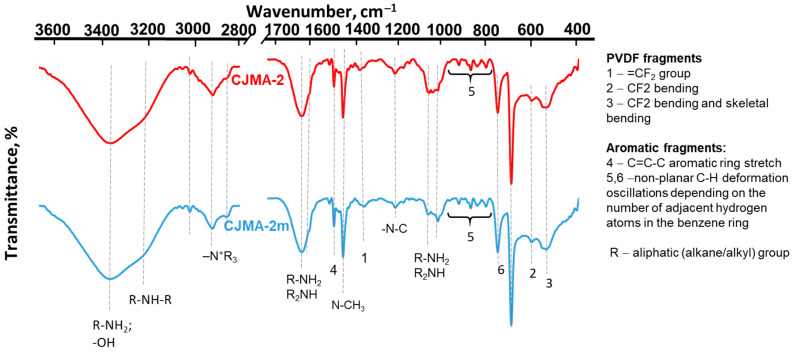
IR spectrum of the CJMA-2 pristine and the CJMA-2m modified membranes.

Figure 3 summarizes the IR spectra of the CJMA-2 pristine and the CJMA-2m modified membranes. In general, these IR spectrums are almost identical. The absorption bands at 1390 cm^−1^, 609 cm^−1^, and 546 cm^−1^ refer to symmetric and asymmetric bending vibrations of the CF_2_ bond. These peaks are characteristic of the membrane matrix, which consists of polyvinylidene fluoride, PVDF [55,56,57].

The region of 700–950 cm^−1^ contains many peaks that correspond to out-of-plane bending vibrations of the C-H bonds of adjacent hydrogen atoms in the benzene ring. In addition, the spectrum contains a low-intensity bond in the region of 1620–1610 cm^−1^ and a peak at 1495 cm^−1^ associated with planar vibrations of the benzene ring [58,59].

With regard to our study, the composition of the AEMs functional (fixed) groups before and after modification is very important. Both IR spectrums indicate valence deformation asymmetric and symmetric vibrations of N-CH_3_ or -N^+^R_3_ bonds. They are detected by the peaks in the region of 2925–2850 cm^−1^ and at 1454 cm^−1^ [60,61]. As with most polymeric AEMs [62,63], a wide band given by free and bound OH groups overlaps the region of 3500–3300 cm^−1^ [64]. Therefore, we cannot confidently attribute the peaks observed in this region to valence bond stretching asymmetric and symmetrical vibrations of primary and secondary amines [59,60,65]. However, the IR spectra have the distinct peaks at 1650–1620 cm^−1^ and at 1075–1020 cm^−1^. The authors of some publications, for example [56,60,64,66,67], attribute them to vibrations of the planar deformation of the groups R-NH_2_ or R_2_NH. Stretching vibrations ν(N–C) of tertiary amino groups are indicated by a distinct peak at 1200 cm^−1^ [68,69].

Clear differences in the IR spectra of the pristine (CJMA-2) and the modified (CJMA-2m) membranes are observed only in the region of 1100–1000 cm^−1^. In the case of the CJMA-2m, the peaks at 1075–1020 cm^−1^, which are characteristic of primary and secondary amines, become less pronounced. In addition, a peak appears in this region at 1027 cm^−1^, which some researchers attribute to the C-N vibrations in the composition of diallylammonium chloride [70,71].

Peaks characteristic of the carboxyl group (1760–1680 cm^−1^) [68,69] were not detected in the CJMA-2m IR spectrum. Apparently, quaternary ammonium groups and the aliphatic matrix of the modifier shield these groups, which is the part of PQ-22 [47]. The reaction to form amides of the RCO–NR_2_ type by the carboxyl groups of the modifier and the weakly basic amines of the membrane requires high temperature [72]. The membrane modification temperature (50 °C) [48] is lower. Therefore, the IR spectrum of the CJMA-2m membrane does not contain peaks (3220–3180 cm^−1^, ~1700 cm^−1^) characteristic of amides.

Thus, the basis of the pristine CJMA-2 membrane is a PVDF ion-exchange matrix. These data are correlate with those reported in [73]. Apparently, cross-linker [74] contains aromatic moieties. Strongly basic quaternary ammonium groups that have aliphatic (–CH_3_) radicals are the fixed groups of the CJMA-2 membrane. In addition, the membrane contains weakly basic fixed groups (tertiary, secondary, and primary amines). Note that the product brochure provided by manufacture only mentions quaternary ammonium groups for this membrane [73].

**Figure 4:**
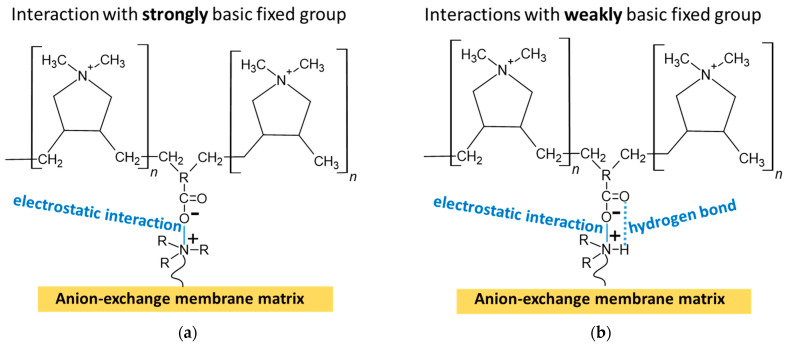
Possible interaction of the modifier, PQ-22, with strongly basic (**a**) and weakly basic (**b**) fixed groups of the anion-exchange membrane. Thin blue solid lines indicate the electrostatic interaction. Dotted blue lines indicate hydrogen bond.

We believe that in the case of quaternary ammonium groups, only electrostatic interactions take place with the modifier, PQ-22 (Figure 4a). In the case of proton-containing weakly basic amino groups, interactions, probably, are similar to those that cause the formation of the “bound species” [41,43,45,46] (Figure 4b). Indeed, it is known [75,76] that the hydrogen bond energy in a salt of carboxylic and amino group usually does not exceed 4–10 kcal/mol. Nevertheless, hydrogen bonds give these salts specific properties that are not the sum of the properties of their constituents.

Thus, electrostatic interactions and hydrogen bonds seem to keep the modifier on the surface and in the bulk of AEM with weakly basic fixed groups for 100 h or more of continuous operation [77]. On the contrary, under the influence of an electric field, PQ-22 (and similar modifiers based on copolymers of diallyldimethylammonium chloride with carboxylic acids) are easily removed from the surface of membranes that contain mainly quaternary ammonium groups [77].

#### 3.1.2. Surface Geometry and Hydrophilicity

**Figure 5:**
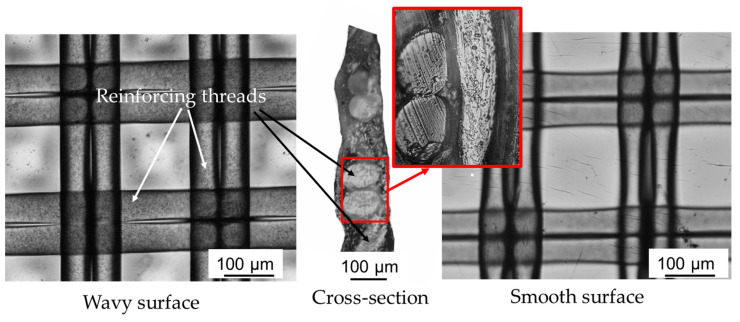
Optical images of the surfaces and cross-section of the wet CJMA-2 membrane.

**Figure 6:**
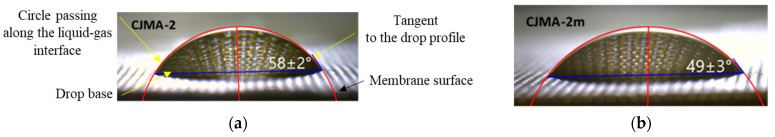
Droplets and contact angles on wavy surfaces of wet pristine (**a**) and modified (**b**) membranes. The membranes were pre-soaked in 0.02 M NaCl solution. The contact angles were measured 20 s after the drop was applied to the membrane surface.

Optical images of the surfaces and cross-sections of the CJMA-2 membrane are shown in Figure 5. Figure 6 contains images of droplets on the surfaces of the studied membranes as well as contact angle values.

The surface of the wet CJMA-2 membrane, into which the reinforcing fabric is pressed, is smoother compared to the opposite more wavy surface (Figure 5). The valleys correspond to the middle of the mesh of the reinforcing cloth. The hills are above the intersection of perpendicular twin threads. The average distance in height between the bottom of the valleys and the tops of the hills (*R_t_* according to [78]) reaches 11.6 ± 4.1 μm and 48.0 ± 3.0 μm for smooth and wavy surfaces, respectively. The distance between the highest points of neighboring hills (280 ± 20 μm) is equal to the mesh pitch size of reinforcing cloth. These data are in good agreement with the results obtained by profilometry [50]. The area of the wavy surface is 6 ± 2% larger than the area of the smoother surface. The average thickness of the wet CJMA-2 found from the optical cross-section is 140 ± 2 μm (Figure 5). The modified membrane has similar characteristics.

Both surfaces of the CJMA-2 membrane are characterized by the same (within the measurement error) values of the contact angle: 58 ± 2°. The wavy surface of the modified CJMA-2m membrane is more hydrophilic compared to the pristine CJMA-2 membrane (Figure 6). The increase in the hydrophilicity of the CJMA-2m is probably a consequence of the increase in the concentration of the positively charged fixed ammonium groups on its surface due to modification. For example, in the case of a similar modification of MA-41P membranes (OOO Shchekinoazot, Pervomaisky, Russia) and Ralex AHM PES (MEGA, Straz Pod Ralskem, Czech Republic), the surface charge increased by 1.5 times in comparison with pristine membranes [47].

It is known that both an increase in surface waviness and an increase in its electric charge contribute to the development of electroconvection, which can increase the useful mass transfer [79,80,81]. Therefore, the wavy surface of the membranes was faced into the desalination compartment (DC) of the electrodialysis cell in further studies.

#### 3.1.3. Ion-Exchange Capacity, Water Uptake, Conductivity

Data that are significant for predicting the transport characteristics of the studied membranes are presented in Table 2.

The ion-exchange capacity, water content of the CJMA-2 membrane, as well as conductivity and effective counterion (Cl^−^) transport number in NaCl solution are comparable to those of other AEMs [82] manufactured by the Hefei Chemjoy Polymer Materials Co. Ltd. A high water content with a relatively low ion-exchange capacity is indicative of weaker cross-linking of the CJMA-2 ion-exchange matrix compared to, for example, an ASE anion-exchange membrane (Astom, Shunan, Japan) [83]. The relatively weak cross-linking of the main chains of the CJMA-2 matrix should reduce steric hindrance in the transport of large hydrated phosphates in this membrane. However, the conductivity of this membrane decreases by a factor of 2.8 when replacing 0.5 M NaCl solution with NaH_2_PO_4_ solution. Note that the ratio of the diffusion coefficients of counterions Cl^−^ and H_2_PO_4_^−^ or HPO_4_^2−^ in an infinitely dilute solution is 2.1 and 2.5 [63], respectively. A more significant decrease in the CJMA-2 conductivity membrane suggests specific interactions of phosphates with weakly basic groups.

The ion-exchange capacity of the CJMA-2m membrane do not differ significantly from those of the CJMA-2 because the acid–base method (see Supplementary Materials) is applied to determine them. This method takes into account both strong basic and weak basic fixed groups of the CJMA-2 membrane. In addition, the modifier only acts on the part (weakly basic) of the fixed groups of the pristine membrane. Within the measurement error, the water content in the modified membrane remains as high as in the pristine membrane. The conductivity of the CJMA-2 m membrane in NaCl solution (pH 5.4) increases 1.3 times compared to the CJMA-2 (Table 2). This fact is an indicator of a decrease in the ion-exchange capacity of the pristine membrane by about 30% due to the deprotonation of weakly basic fixed groups. The ratio of the conductivities of the CJMA-2m and the CJMA-2 membranes increases to 1.9 in Na_x_H_(3−x)_PO_4_ solution with pH 4.5 ± 0.1. This growth seems to be promoted by shielding of the weakly basic groups of the CJMA-2 membrane by the quaternary ammonium groups of the modifier (Figure 4b). This phenomenon and its implications will be discussed in Section 3.3.

### 3.2. Electrochemical Characteristics of the Studied Membranes in NaCl Solutions

**Figure 7:**
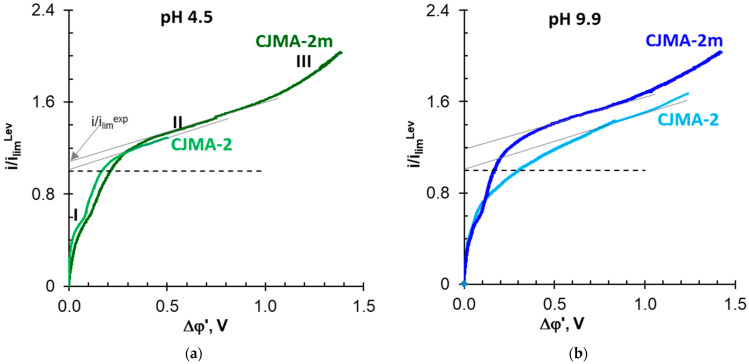
Current–voltage curves of the CJMA-2 pristine and the CJMA-2m modified membranes in 0.02 M NaCl solutions with pH 4.5 ± 0.1 (**a**) and 9.9 ± 0.1 (**b**). The dashed line corresponds to the value *i/i_lim_^Lev^* = 1.0. The gray auxiliary lines show the procedure for i*_lim_^exp^* determining.

Figure 7 shows the current–voltage curves (CVCs) of the CJMA-2 and the CJMA-2m membranes obtained in 0.02 M NaCl solutions with pH 4.5 ± 0.1 and 9.9 ± 0.1. In general, the electrochemical behavior of studied membrane is in good agreement with the results obtained earlier [47] for other AEMs in NaCl solutions. In an acidified (pH 4.5 ± 0.1) solution, the shapes of the CVCs of pristine and modified membranes almost do not differ from each other. The initial sections *I* of the CVCs (Figure 7a) contain a small “distortion” near *i/i_lim_^Lev^* = 0.6. Its appearance is caused probably by the reaching of the limiting state in stagnant zones [34] at the “valleys” of the CJMA-2 and the CJMA-2m surface. At the current density close to *i_lim_^Lev^*, the limiting state extends to the entire wavy surface of the CJMA-2 membrane. Limitation of the electrodiffusion delivery of the electrolyte from the bulk to the AEM/depleted solution interface manifests itself in the formation of an inclined plateau II [84]. The equilibrium electroconvection control the slope and the “length” of the section II [85]. Development of the unsteady Rubinstein–Zaltzman electroconvection [86] and water splitting affect the slope of the overlimiting section III. Indeed, most of the weakly basic groups on the surface of the CJMA-2 are protonated in an acidified solution [83]. Thus, most of them take part in the transfer of counterions. Modification using PQ-22 contributes to an increase in the electric charge [47] CJMA-2m compared to the CJMA-2 membrane (Figure 4b). This growth stimulates electroosmosis of the first kind [67,85,86], which contributes to an increase in *i_lim_^exp^* by 8% compared to the CJMA-2 membrane. Note that an increase in the charge of the CJMA-2m surface is accompanied by an increase in its hydrophilicity (Figure 6b), which restrains the liquid slip [87] along the CJMA-2m surface. Therefore, the *i_lim_^exp^* increment is not significant compared to the CJMA-2 membrane.

**Figure 8:**
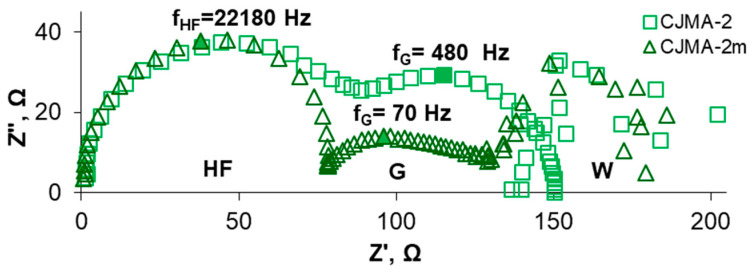
Electrochemical impedance spectra of the CJMA-2 pristine and the CJMA-2m modified membranes in 0.02 M NaCl solution with pH 4.5 ± 0.1. The spectra were obtained at *i = 1.5i_lim_^Lev^*. The letters denote the high-frequency arch (HF), as well as the Warburg (W) and Gerischer (G) arches. The frequency *f_G_* is shown.

Nevertheless, the modification leads to a suppression in water splitting at the interface AEM/depleted solution. For example, the difference at the inlet and outlet of the desalination compartment is −0.44 (CJMA-2) and −0.17 (CJMA-2m) if i/i_lim_^Lev^ = 1.5. The results of electrochemical impedance spectroscopy (Figure 8) provide more reliable information concerning water splitting. The Gerischer arch on the CJMA-2m impedance spectrum is less pronounced compared to the pristine one. As estimated using Equation (5) [36,54], the effective rate constant χ of water splitting decreases by almost seven times: from 1740 s^−1^ (CJMA-2) to 250 s^−1^ (CJMA-2m). Suppression of water splitting at the surface of the modified membrane is caused by the low catalytic activity of PQ-22 quaternary ammonium groups to generate H^+^, OH^−^ ions [88,89]. On the contrary, the weakly basic fixed amino groups have very high activity in this reaction [88,89].

In the solution with pH 9, the shape and main parameters of the CJMA-2m CVC (Figure 7b) practically do not change in comparison with the acidified (pH 4.5) solution. The quaternary ammonium groups of PQ-22, which shield the weakly basic fixed groups, ensure this behavior of the modified membrane. On the contrary, weakly basic amino groups become fully deprotonated in the CJMA2 membrane and do not participate in the transport of counterions.

This conclusion is theoretically and experimentally substantiated in the Ref. [90], taking into account the equilibrium constants of the protonation–deprotonation reactions for the primary and secondary amino groups (10^−5^–10^−3^ cm^3^ mmol^−1^) and for the tertiary amino groups (10^−8^–10^−7^ cm^3^ mmol^−1^) [91,92,93]. As estimated [92], the actual decrease in the ion-exchange capacity causes an increase in electrical resistance and suppression of AEM selectivity. In addition, the loss of electric charge by weakly basic fixed groups on the CJMA-2 surface should suppress the development of electroconvection by electroosmosis of the first kind mechanism. The superposition of these phenomena causes a decrease of 20% *i_lim_^exp^* and overlimiting currents in the case of the CJMA-2 compared to the CJMA-2m membrane (Figure 7b). Moreover, the section I of the CVC transforms into section II at higher potential drops than in the acidified solution (Figure 7).

### 3.3. Electrochemical Characteristics of the Studied Membranes in Na_x_H_(3−x)_PO_4_ Solutions

**Figure 9:**
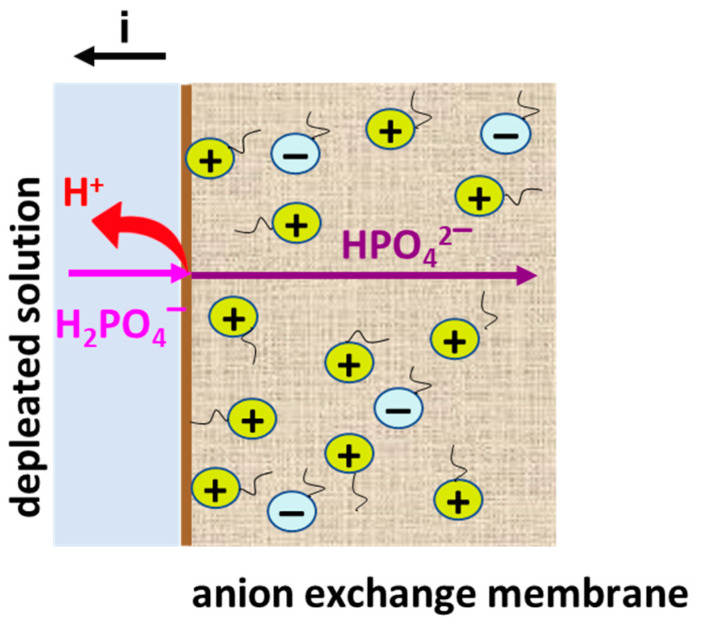
Schematic representation of the implementation of the “acid dissociation” mechanism in the system NaH_2_PO_4_ solution/anion exchange membrane with strongly basic fixed groups.

The AEM’s electrochemical behavior becomes more complex in Na_x_H_(3−x)_PO_4_ solution due to the involvement of phosphates in proton transfer reactions. Solutions (pH 4.5 ± 0.1) that contain more than 99% singly charged phosphates (Table 1) are the most studied [31,34,36]. In the case of AEMs containing only quaternary ammonium groups, the “acid dissociation” mechanism (Figure 9) controls a CVC shape. According this mechanism, singly charged H_2_PO_4_^−^ enter the AEM under the influence of an electric field and dissociate into a proton and a doubly charged HPO_4_^2−^ anion. The protons are the coions. Therefore, Donnan exclusion pushes them into the depleted solution of the desalination compartment. Anions HPO_4_^2−^ are transferred in the membrane to the enriched solution of the concentration compartment. This phenomenon takes place at any current density, but increases at I ≥ i_lim_^Lev^.

**Figure 10:**
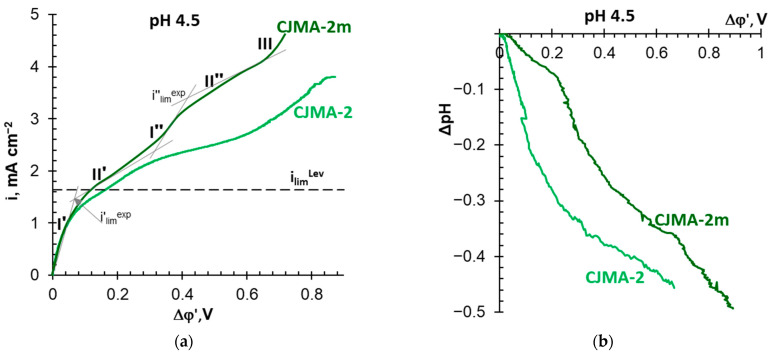
Current–voltage curves of the CJMA-2 pristine and the CJMA-2m modified membranes in 0.02 M Na_x_H_(3−x)_PO_4_ solution with pH 4.5 ± 0.1 (**a**), as well as the pH difference of the solution at the inlet and outlet of the desalination compartment, obtained simultaneously with CVCs measurement (**b**). The dashed line indicates the value of the theoretical limiting current, i_lim_^Lev^ = 1.62 mA cm^−2^.

The shape of the CVCs (Figure 10a) and the behavior of the CJMA-2m membrane, in which the modifier shields weakly basic groups, do not differ from the shape of CVCs obtained for other AEMs with quaternary ammonium groups. ASE, AMX, AMX-sb (manufactured by Astom, Japan), CJMA-3 (manufactured by Hefei Chemjoy Polymer Materials Co. Ltd., China), MA-41, MA-41P (manufactured by OOO Shchekinoazot, Russia), and IONSEP-HC-A (manufactured by Hidrodex^®^, Cotia, São Paulo, Brazil) are among them [19,31,32,34,83]. Some characteristics of these membranes as well as CVCs are presented in the Supplementary Materials.

Indeed, at the vicinity of i′*_lim_^exp^*, just as in the case of the NaCl solution, the electrolyte concentration in the depleted solution near the CJMA-2m surface becomes negligible compared to the bulk solution. This state corresponds to *i*′*_lim_^exp^*, which is found by the intersection point of the tangents to sections I′ and II′ of the CVC. Dilution of the depleted solution enhances the Donnan exclusion of protons from the CJMA-2m membrane and the conversion of singly charged phosphoric acid anions into doubly charged ones. The appearance of additional charge carriers in the solution at the AEM surface and the doubling of the electric charge of the counterions inside the membrane causes an increase in the current density in the section I″. Membrane saturation with HPO_4_^2−^ anions is characterized by the appearance of an inclined plateau II″ and one more experimental current *i*″*_lim_^exp^*. Moreover, the value of *i*″*_lim_^exp^* doubles in comparison with *i*″*_lim_^exp^*. Water splitting and unsteady electroconvection mainly control the conductivity of the membrane system in section III, as in the case of NaCl solutions (Section 3.2).

**Figure 11:**
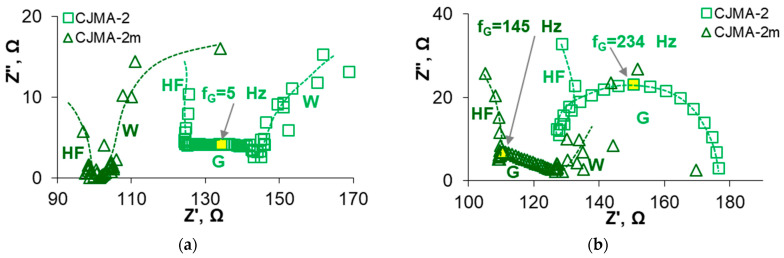
Electrochemical impedance spectra of the CJMA-2 pristine and the CJMA-2m modified membranes in 0.02 M Na_x_H_(3−x)_PO_4_ solution with pH 4.5 obtained at *i =* 1.3 *i_lim_^Lev^* (**a**) and *i =* 2.0 *i_lim_^Lev^* (**b**). HF, G, and W letters denote the high frequency, Gerischer, and Warburg arches, respectively. The dotted lines are a guide to the eyes.

Acidification of the desalted solution at a potential drop below 0.2 V is an indicator of the proton generation by the CJMA-2m membrane due to the “acidic dissociation” mechanism (Figure 10b). An increase in the slope of the ΔpH–Δφ′ curve at Δφ′ > 0.2 V characterizes the increase in the Donnan exclusion of protons from the membrane in overlimiting modes. The slope of the ΔpH–Δφ′ curve becomes even more significant at Δφ′ > 0.7 V (section III, CVC), when water splitting supplements the “acid dissociation”. Accordingly, the Gerischer arch appears on the impedance spectra of the CJMA-2m membrane (Figure 11) only at current densities (potential drops) that correspond to section III (Figure 10a). At *i =* 2.0 *i_lim_^Lev^*, the value of the effective rate constant of the proton generation reaction, χ, is 530 s^−1^ for the CJMA-2m membrane. Moreover, these values turn out to be an order of magnitude lower than the value (4530 s^−1^) found earlier under the same conditions for the AMX membrane (Astom, Japan) [36].

As rightly pointed out by Rotta, et al. [32], diffusion of new charge carriers from the chemical reaction zone into the membrane and depleted solution contributes to the Gerischer arc. Therefore, the chemical nature of the matrix of the studied AEMs, the structure of their volume and surface, as well as the concentration and localization of fixed groups can affect the parameters of the Gerischer arc, and accordingly, the values of the effective rate constant of the chemical reaction. In addition, AMX could contain a number of weakly basic fixed groups that accumulate during storage and operation of this membrane [82]. Note that in the case of solutions of salts of some polybasic acids, for example, tartaric, citric, or oxalic [35,36], another Gerischer sub-arch appears in the mid-frequency region of the spectrum. This sub-arch is characterized by lower frequencies at the maximum point, and accordingly, lower values of χ compared with water splitting. We believe that this arch corresponds to proton generation reactions by the “acid dissociation” mechanism [36]. In the case of acidic (pH 4–5) solutions of Na_x_H_(3−x)_PO_4_, such a sub-arc was not found [32,36], apparently due to the high rate of the H_2_PO_4_^−^→H^+^+ HPO_4_^2−^ reaction.

It is of note that impedance spectra allow estimation of AEM resistance and adjacent solutions at the point of intersection of the high-frequency arch with the Z′ axis [94]. For the CJMA-2 and the CJMA-2m membranes, the difference in these resistances is 5 Ω at i = 0 (Figure 11a), but increases to 25 Ω at i = 1.3 i_lim_^Lev^ (Figure 11b).

**Figure 12:**
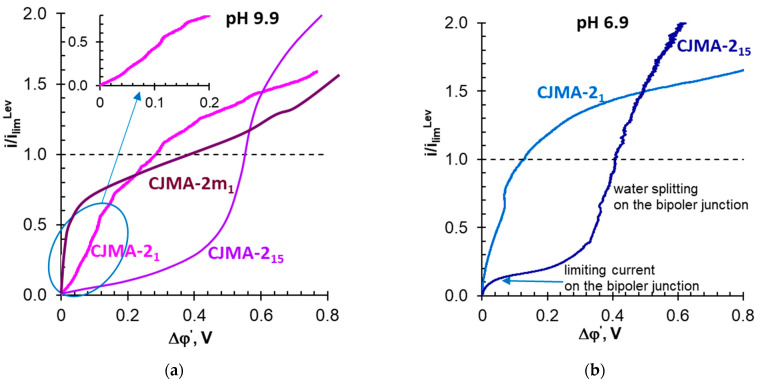
Current–voltage curves of the CJMA-2 and the CJMA-2m membranes in 0.02 M Na_x_H_(3−x)_PO_4_ solutions with pH 9.9 ± 0.1 (**a**) and 6.9 ± 0.1 (**b**). Indexes 1 and 15 correspond to the duration (in hours) of membrane operation in an electric field before the measurements. The dashed line corresponds to *i = i_lim_^Lev^*.

Current–voltage curve of the CJMA-2m membrane in a Na_x_H_(3−x)_PO_4_ solution with pH 9.9 ± 0.1 has one plateau (Figure 12a). This CVC shape is similar to the curves shown in Figure 10a and obtained in many studies for strong electrolytes (NaCl, Na_2_SO_4_, etc.) [35,47,53,54,66]. Similar curves were obtained earlier in Na_x_H_(3−x)_PO_4_ solutions with pH ≥ 9 for membranes (AX, ASE, CJMA-3, etc.) containing mainly quaternary ammonium groups [31,83]. Indeed, the solution contains over 99% Na_2_HPO_4_ (Table 1). The pseudo-unimolecular rate constant of the rate-limiting step of the HPO_4_^2−^ ↔ PO_4_^3−^ + H^+^ reaction is very low, 5·10^−3^ s^−1^ [33]. Therefore, a relatively high potential drop is needed for the conversion of H_2_PO_4_^−^ anions into PO_4_^3−^ anions due to the “acid dissociation” mechanism.

Regarding the CJMA-2, membrane, even in an acidic solution (pH 4.5 ± 0.1), higher potential drops correspond to the given current densities (Figure 10a). In addition, the value of *i*″*_lim_^exp^* is 1.5 times lower in the case of the CJMA-2 membrane compared to the modified one (Figure 10a). The generation of protons is significantly enhanced (Figure 10b). The Gerischer arch appears on the electrochemical impedance spectrum at a slight excess of i′_lim_^exp^ (Figure 11a) and absorbs the Warburg arch at i ≥ i″_lim_^exp^. The χ values (18 s^−1^ at *i =* 1.3 *i_lim_^Lev^*) are of the same order as in the case of the ONSEP-HC-A membrane (Hidrodex^®^,Cotia, São Paulo, Brazil) studied in 0.001 M Na_x_H_(3−x)_PO_4_ solution with pH 5 [32]. At *i =* 2.0 *i_lim_^Lev^*, the values of *f_G_* and, respectively, the effective rate constant of the proton generation reaction for the CJMA-2 membrane becomes 1.6 higher compared to the CJMA-2m membrane (Figure 11b).

In Na_x_H_(3−x)_PO_4_ solution with pH 9.9 ± 0.1, a small inclined plateau appears in the initial section of the CJMA-2 current–voltage curve. The length of the plateau increases with the duration of the CJMA-2 membrane operation in an electric field (Figure 12a) and the CVC shape becomes similar to that of a bipolar membrane [95]. CVC acquires a similar form during long-term operation of the CJMA-2 membrane in 0.02 M Na_x_H_(3−x)_PO_4_ solution with pH 6.9 ± 0.1 (Figure 12b). Note that, under similar conditions, the CVCs of AEMs with strong basic groups have two plateaus [31,32,83]. These CVCs are similar to those described in [83] for the CJMA-6 membrane (Hefei Chemjoy Polymer Materials Co., Ltd., Hefei, China), which contains a mixture of strongly and weakly basic fixed groups and studied alkaline solutions.

Moreover, general for the “fresh” samples of the CJMA-2 and the CJMA-6 membranes [83] is the alkalization of Na_x_H_(3−x)_PO_4_ solution during obtaining CVCs. Proton generation increases with increasing duration of AEM operation in an electric field. After 2–3 h of continuous operation, acidification becomes more significant than in the case of membranes containing predominantly quaternary ammonium groups.

Thus, the CJMA-2 membrane with a mixture of strongly and weakly basic fixed groups differs from the CJMA-2m membrane, in which a modifier shields weakly basic groups, as follows. It has a higher conductivity in a phosphate containing solution.

The fundamental differences in the behavior of the CJMA-2 and the CJMAm-2 membranes can be explained using the following hypothesis:

The weakly basic fixed groups of AEMs (primary and secondary amines) enter into specific interactions with proton-containing phosphoric acid anions. These interactions seem to be similar to those [15,26,27,28,29,30,39,40,41,42,43,44], which were already discussed in the Introduction. According to the electrostatic/hydrogen bond switching model, which describes such interactions [43,44,45,46], proton transfer reactions contribute to the formation of a “bound species” involving the proton-containing species of oxoacids and amino groups. Equations (11)–(15) describe schematically these interactions in the case of primary amines:

H_2_O ↔ OH^−^ + H^+^ (11)

H_2_PO_4_^−^ ↔ HPO_4_^2−^ + H^+^ (12)

R–N^+^H_3_ ↔ R–NH_2_ + H^+^ (13)

R–N^+^H_3_ + H_2_PO_4_^−^ ↔ R–[(NH_3_)H_2_PO_4_]^0^ (14)

R–N^+^H_3_ + HPO_4_^2−^ ↔ R–[(NH_3_)HPO_4_]^−.^ (15)

In the case of the participation of HPO_4_^2−^ anions, the binding constant for the “bound species” is one order of magnitude higher compared with species formed by doubly charged anions of oxalate or sulfuric acids [41]. Hydrogen bonds between the hydrogen atoms of amines and the oxygen atoms of phosphates facilitate proton transfer reactions between these species [45]. Therefore, the following interactions are possible:

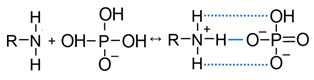
(16)

A thin blue solid line indicates the electrostatic interactions; a dotted blue line indicates the hydrogen bonds of the “bound species” on the right side of Equation (16). Estimates by Equations (11)–(15) [46] predict the predominance of the mole fraction of [(NH_3_)H_2_PO_4_]^0^ and R[(NH_3_)HPO_4_]^−^ species at pH from 4 to 10 if the solution contained 0.02 M phosphates. Species RN^+^H_3_ predominate at pH < 4. Species RNH_2_ dominate at pH > 10.

**Figure 13:**
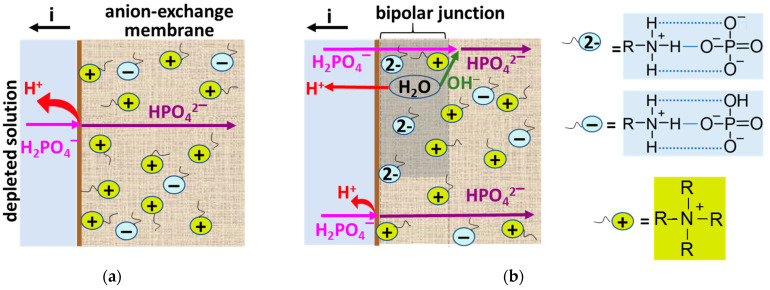
Assumed schemes of ion transport and proton generation in the case of acidified (**a**) and alkaline (**b**) Na_x_H_(3−x)_PO_4_ solutions. The anion exchange membrane contains a mixture of strongly and weakly basic fixed amino groups.

Recall that the equilibrium constants of primary and secondary aliphatic amines protonation–deprotonation are 10^−3^–10^−5^ cm^3^ mmol^−1^ [91,92,93]. Thus, in the case of Na_x_H_(3−x)_PO_4_ solutions with pH 4.5 ± 0.1 protonated RN^+^H_3_, weak acid fixed groups are located mainly on the CJMA-2 surface. These groups actively participate in water splitting (Figure 10b and Figure 11), as in the case of NaCl solutions [88,89] (Figure 8). Inside the AEMs, pH is shifted by three or more units to the alkaline region due to the Donnan exclusion of H^+^ ions [31]. These pH values are favorable for the formation of neutral (R[(NH_3_)H_2_PO_4_]^0^) and negative (R[(NH_3_)HPO_4_]^−^) “bound species” (Figure 13a) that reduce the ion-exchange capacity and inhibit the transfer of phosphates in AEM. Both phenomena contribute to decreasing the conductivity of the CJMA-2 membrane (Table 2). In addition, the exclusion of coions (protons) from the membrane according to Donnan is reduced, and “acid dissociation” is suppressed (Figure 10a). Hence, the concentration of protons in the depleted diffusion layer decreases, and accordingly, its resistance increases (Figure 11). Therefore, the Δφ′ values increase for a given current on the CVC of the CJMA-2 membrane (Figure 10a). Phosphates do not form “bound species” with quaternary groups of the PQ-22 modifier, which shields weakly basic fixed groups. Therefore, the characteristics of the CJMA-2m membrane are better in all respects compared to the CJMA-2.

In Na_x_H_(3−x)_PO_4_ solutions with pH 6.9 ± 0.1 and 9.9 ± 0.2, the weakly basic fixed amino groups of the CJMA-2, apparently, are mostly deprotonated on the membrane surface and uniquely deprotonated in AEM volume. The volume of AEMs contains a high proportion of doubly charged phosphates. Therefore, the formation of a negatively charged bound species is the most probable (Figure 13a). Just as in an acidified Na_x_H_(3−x)_PO_4_ solution, this phenomenon suppresses “acid dissociation”. This suppression is expressed in the presence of only one sloping plateau in the CVC obtained in Na_x_H_(3−x)_PO_4_ solutions with pH 6.9 ± 0.1 (Figure 12a).

The triply charged phosphates concentration is very low in AEMs in the case of Na_x_H_(3−x)_PO_4_ solutions with pH 6.9 ± 0.1 and 9.9 ± 0.2. This is evidenced by estimates made taking into account the equilibrium protonation–deprotonation constants [31]. However, the acceleration of the proton removal from AEM due to the electric field, apparently, shifts the equilibrium in favor of PO_4_^3−^ formation. These anions form doubly charged type R[(NH_3_)PO_4_]^2−^ species with deprotonated primary and secondary amines. Enhancing Donnan exclusion by reducing the concentration of electrolyte in the depleted solution promotes their formation at the interface (Figure 13b). The double charge species-rich layer and generally positive AEM volume form a bipolar junction, as in the case of bipolar membranes [93]. The formation of such a bipolar junction takes time. The wider the negatively charged layer near the surface of the AEM, the longer the plateau observed on the CVC becomes (Figure 12).

After turning on the current, cations and anions leave the bipolar junction, forming a depletion layer in its vicinity. In the case of Na_x_H_(3−x)_PO_4_ solutions with pH 6.9 ± 0.1, a small number of R[(NH_3_)PO_4_]^2−^ species seem to form. Therefore, the negative layer of the bipolar junction is “loose” and contains some coions. Thus, its depletion in ions takes place at a well-observed current (Figure 12a). The high (9.9 ± 0.2) pH value of Na_x_H_(3−x)_PO_4_ solutions promotes the formation of R[(NH_3_)PO_4_]^2−^ species. The high (9.9 ± 0.2) pH value of Na_x_H_(3−x)_PO_4_ solutions promotes the formation of a denser bipolar junction. A small number of coions in its negative layer manifests itself in a low value of the limiting current and the achievement of high potential drops before the onset of water splitting (Figure 12).

Unfortunately, we did not obtain the electrochemical impedance spectra of the CJMA-2 and the CJMA-2m membranes in Na_x_H_(3−x)_PO_4_ solutions with 6.9 and 9.9 due to the limitation of the electrochemical Autolab complex in carrying out measurements at high values of the potential drop. However, Rotta et al. [32] observed two Gericsher sub-arches in dilute Na_x_H_(3−x)_PO_4_ solution with pH 7.2. We believe that one of the sub-arcs corresponds to the water splitting at the AEM/depleted solution interface, and the other sub-arc possibly characterizes the water splitting at the bipolar junction.

This discussion provides the key to interpreting the results of electrodialysis desalting of phosphate-containing solutions using the pristine and modified membranes.

### 3.4. Batch Electrodialysis of Na_x_H_(3−x)_PO_4_ Solution

The initial concentration of Na_x_H_(3−x)_PO_4_ solution with pH 4.5 ± 0.1 was 0.03 M. The experiments were carried out at current densities equal to 1.63, 2.46, 3.25, and 3.75 mA cm^2^. Table 3 contains the relationships i/i_lim_^Lev^ at the beginning and at the end of each electrodialysis run, as well as the ED performance at the 40% degree of recovery of pentavalent phosphorus from the diluate stream. The values of limiting current were calculated by the Equation (5).

**Figure 14:**
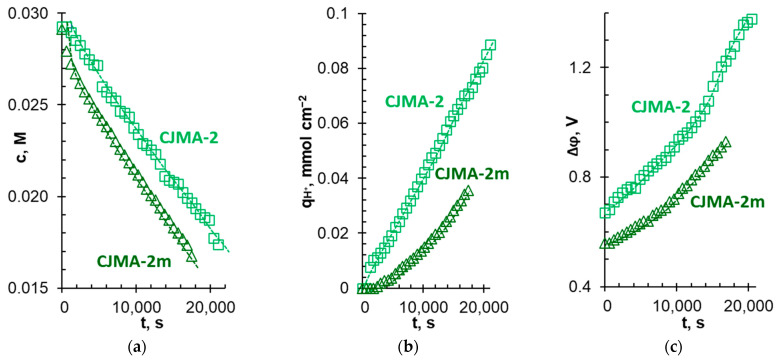
Electrolyte concentrations (**a**) and the number of protons coming from the membrane into the diluate stream (**b**), as well as potential drops measured by Luggin capillaries on the studied AEM and adjacent layers of the solution (**c**) vs. the duration of the ED desalination of 0.03 M Na_x_H_(3−x)_PO_4_ with pH 4.5 ± 0.1. Electrodialysis was carried out at 1.63 mA cm^−2^.

**Figure 15:**
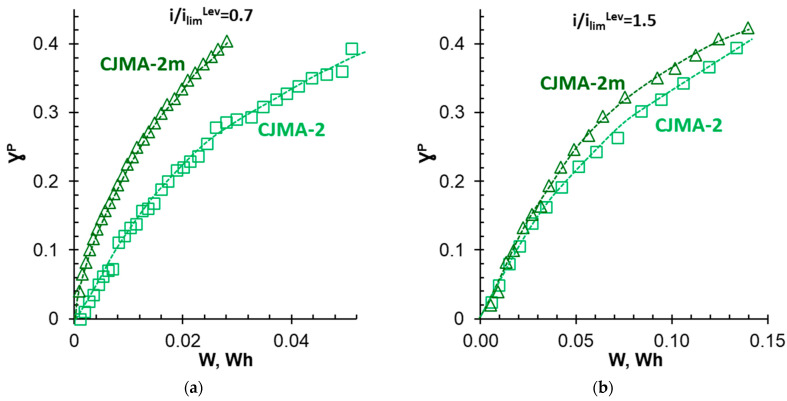
The degree of recovery of pentavalent phosphorus from the diluate stream vs. energy consumption during electrodialysis at current densities of 1.63 mA cm^−2^ (**a**) and 3.75 mA cm^−2^ (**b**).

**Figure 16:**
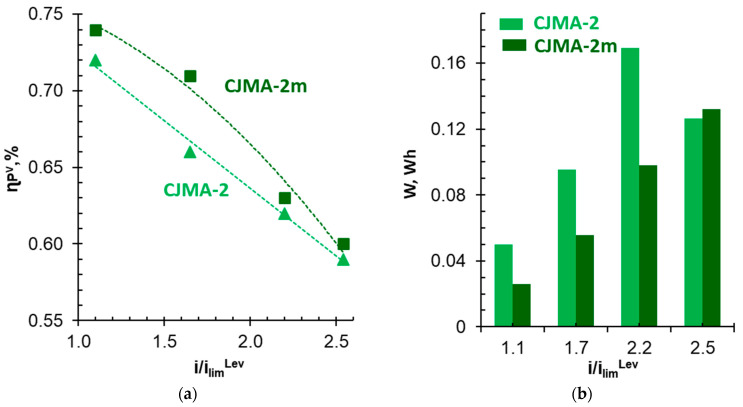
Current efficiency for pentavalent phosphorus (**a**) and energy consumption (**b**). Given current density normalized to the theoretical limiting current. The data correspond to 40% the degree of phosphates recovery from the diluate stream.

Figure 14, Figure 15 and Figure 16 show some characteristics of the ED performance depending on the duration of electrodialysis. Data were obtained at a current density of 1.63 mA cm^−2^, which corresponds to 0.66 i_lim_^Lev^ at the beginning of the ED.

The concentration of phosphates in the diluate stream decreases faster (Figure 14a), and the number of protons coming from the membrane into the diluate stream decreases (Figure 14b) if the CJMA-2m modified membrane replaces the CJMA-2 pristine membrane in the membrane stack. In addition, the potential drop in the case of the CJMA-2m membrane is lower than in the case of the CJMA-2 membrane (Figure 14c). Improvement in the ED performance leads to a significant reduction in energy consumption to achieve a given degree of P^V^ recovery if the membrane stack contains a modified membrane (Figure 15a). Desalination of a 0.03 M solution by 40% is 1.3 times faster and energy consumption is reduced by 1.9 times when using a modified membrane compared to a pristine membrane (Table 3).

In the case of using both membranes studied, an increase in the given current density leads to a reduction in the duration of electrodialysis to achieve the desired (40%) degree of recovery of pentavalent phosphorus (Table 3). However, current efficiency (${ɳ}^{P^{V}}$) decreases (Figure 16, Table 3). The linear dependence of ${ɳ}^{P^{V}}$ upon i/i_lim_^Lev^ is observed for the CJMA-2 membrane in the investigated range of current densities. In the case of the modified membrane, the ${ɳ}^{P^{V}}$ values depend less on the current density if i ≤ 1.0 i_lim_^Lev^ at the beginning of the ED. A significant drop in current efficiency is observed at i > 1.0 i_lim_^Lev^. The ${ɳ}^{P^{V}}$ values for both membranes become the same within the measurement error at i ≥ 1.5 i_lim_^Lev^. However, at any current density, the potential drops measured on the CJMA-2m membrane are smaller compared to the pristine CJMA-2 membrane (Table 3). This difference rapidly decreases at i ≥ 1.5 i_lim_^Lev^. Nevertheless, even at these current modes, there is a small gain in energy consumption to achieve a given the degree of phosphate recovery when using the CJMA-2m compared to the CJMA-2 membrane (Figure 15b and Figure 16b). The greatest difference in energy consumption (0.06 Wh) is achieved at i = 1.3 i_lim_^Lev^ (Figure 16b).

The presented experimental data (Figure 14, Figure 15 and Figure 16, Table 3) demonstrate a clear advantage in the use of the CJMA-2m modified membrane in comparison with the CJMA-2 pristine membrane, which contains a mixture of strongly basic and weakly basic fixed amino groups. However, the influence of the current mode on the ED performance requires discussion. In the ED run (Figure 14), when the initial and final values of i/i_lim_^Lev^ are 0.66 and 1.10, respectively, the benefits of the CJMA-2m membrane are mainly governed by phenomena in its volume. The low catalytic activity of quaternary ammonium groups prevents the development of intense water splitting in these conditions.

At higher initial (and final) values of i/i_lim_^Lev^, water splitting increasingly affects ED performance by increasing the proportion of multiply charged phosphoric acid anions that are transported in the membranes, and accordingly, reducing the current efficiency. Indeed, at given current densities 3.25 and 3.75 mA cm^−2^, most of the time ED desalination is carried out under favorable conditions for water splitting, i/i_lim_^Lev^ ≥ 2 [31]. Under these conditions, membrane modification does not inhibit water splitting because the high electric field strength at the AEM/diluted solution interface has a greater effect on the generation of H^+^, OH^−^ ions than the low catalytic activity of the quaternary ammonium groups [88,89] on the CJMA-2m surface.

Another reason for the decrease in the advantage of the CJMA-2m membrane with increasing current density is the possible removal of PQ-22 from the surface of the modified membrane in intense current modes [77]. In this case, the intensity of water splitting on the surfaces of the modified and original membranes gradually becomes the same. At the same time, the volume of the membrane, apparently, is less susceptible to the removal of the modifier than its surface. Therefore, the higher conductivity of the modified membrane provides a reduction in energy consumption compared to CJMA-2 in a wide range of current densities.

Note that too intense generation of H^+^, OH^−^ ions (ED run with initial value of i/i_lim_^Lev^ equal to 1.54) causes intensive transport of hydroxyl ions in the CJMA-2m membrane. A high concentration of these ions in the membrane volume can cause degradation of the modifier and the partial transformation of quaternary ammonium bases into weakly basic amino fixed groups, as was repeatedly observed for other membranes [82,90].

## 4. Conclusions

Weakly basic primary and secondary amines are extremely undesirable in the composition of fixed groups of the anion exchange membranes (CJMA-2), which are used in the ED processing of phosphate-containing solutions. Apparently, weakly basic fixed groups form the “bound species” with proton-containing phosphoric acid anions. These R[(NH_3_)H_2_PO_4_]^0^ and R[(NH_3_)HPO_4_] “bound species” acquire a neutral or negative electric charge if external solution contains mainly single-charged HPO_4_^−^ anions.

The ion-exchange material of the CJMA-2 membrane is enriched with R[(NH_3_)PO_4_]^2−^ doubly charged “bound species” at the boundary with the depleted solution if the external solution contains a mixture of singly and doubly charged phosphoric acid anions (pH 6.9) or mainly doubly charged anions (pH 9.9). These negatively charged “bound species” form a bipolar junction with an overall positively charged volume of the anion-exchange membrane. Moreover, alkalization of the external solution, an increase in the current density and duration of operation of the CJMA-2 membrane in an electric field, contribute to the expansion of the bipolar junction. As a result, the current–voltage curves of the membrane become more and more similar to those characteristic of bipolar membranes, the electrical resistance of the CJMA-2 membrane increases, and proton generation enhances significantly.

Modification of the CJMA-2 membrane with polyquaternium-22 (PQ-22), which contains carboxyl groups and quaternary ammonium groups in the ratio of 1:2, makes its behavior close to that of anion-exchange membranes with quaternary ammonium groups. Apparently, the quaternary ammonium bases of the modifier shield the weakly basic groups of the CJMA-2 membrane, preventing the formation of bound species.

The effect of the modifier is manifested in an increase in the conductivity of the CJMA-2m compared to the CJMA-2 by a factor of 1.1 (0.1 M Na_x_H_(3−x)_PO_4_ solution with pH 4.5). In addition, the experimental limiting current increases by a factor of 1.3 in a 0.02 M Na_x_H_(3−x)_PO_4_ solution with pH 4.5. The ED recovery of 40% phosphates in batch hydrodynamic regime is 1.3 times faster, and energy consumption is reduced by 1.9 times when using the CJMA-2m modified membrane compared to the CJMA-2 pristine membrane. The best results are achieved when electrodialysis is carried out at current densities (i < 2 il_im_^Lev^) that are unfavorable for the development of water splitting. Increasing the current density reduces the difference in the behavior of the CJMA-2m modified and the CJMA-2 pristine membranes. However, a decrease in energy consumption for electrodialysis takes place even in very intensive current modes.

The results of the study convince us of the usefulness of using anion-exchange membranes only with strongly basic fixed groups for ED processing of phosphate-containing solutions. The shielding of weakly basic fixed groups with quaternary ammonium groups of modifiers is also very promising. These conclusions are probably relevant for the ED processing of solutions with any anions of polybasic acids. We are going to clarify this in future studies. In addition, we plan to study in more detail the behavior of the modifier in alkaline phosphate-containing solutions.

## Figures and Tables

**Table 1:** Component composition of the studied Na_x_H_(3−x)_PO_4_ solutions depending on their pH.

pH of the Solution	Solution Composition in Mole Fractions, %		
	NaH_2_PO_4_	Na_2_HPO_4_	Na_3_PO_4_
4.5	99.40	0.59	2.66 × 10^−9^
6.6	79.80	20.19	3.38 × 10^−5^
9.9	0.17	99.50	0.33

**Table 2:** Some characteristics of the studied membrane.

Membrane	Ion-Exchange Capacity, mmol/g_dry_	Water Content, g_H2O_/g_dry_, %	* Conductivity, mS cm^−1^ in 0.5 M Solution		* t_1_ in 0.5 M NaCl Solution
			NaCl (pH 5.4)	NaH_2_PO_4_ (pH 4.5)	
CJMA-2	0.9 ± 0.1 [73]	35 ± 5 [73]	2.3 ± 0.1	0.8 ± 0.1	0.97 ± 0.1
CJMA-2m	1.0 ± 0.1	35 ± 5	3.0 ± 0.1	1.5 ± 0.1	0.97 ± 0.1

* The concentration dependences of the conductivity and effective transport numbers of AEMs are presented in the Supplementary Materials.

**Table 3:** Some characteristics of the electrodialysis process at the 40% degree of recovery of pentavalent phosphorus (P^V^) from the diluate stream.

i, mA cm^−2^	i/i_lim_^Lev^		Membrane	ED Duration, s	qH^+^, mol m^−2^	Δφ, V	${ɳ}^{P^{V}}$	W 10^3^, Wh
	γ_P_ = 0%	γ_P_ = 40%						
1.63	0.66	1.10	CJMA-2	20,000	0.83	1.38	0.72	50
			CJMA-2m	15,600	0.30	0.93	0.74	26
2.46	1.00	1.65	CJMA-2	14,100	0.90	2.56	0.66	95
			CJMA-2m	13,500	0.82	1.60	0.71	56
3.25	1.30	2.20	CJMA-2	12,300	1.25	3.65	0.62	169
			CJMA-2m	11,400	0.10	2.54	0.63	98
3.75	1.52	2.54	CJMA-2	9300	0.13	3.34	0.59	126
			CJMA-2m	11,400	0.13	3.16	0.60	130

## Data Availability

Not applicable.

## Supplementary Materials

The following supporting information can be downloaded at: https://www.mdpi.com/article/10.3390/membranes13070647/s1, Figure S1: SEM images of (a) surfaces and cross-section of the heterogeneous MC-40 membrane (b). The heterogeneous membrane MA-41 has a structure similar to that of MC-40; Figure S2: The distribution of species of the orthophosphoric acid (in mole fractions) vs. the pH of the solution; Figure S3: Cross-section of the ion exchange membrane volume in the framework of microheterogeneous model; Figure S4: Schematic of the unit for measuring the diffusion permeability of membranes: (1) two-compartment cell, (2) membrane under study, (3, 4) flow-through compartments of cell 1, (5) tank with distilled water, (6) tank with an electrolyte solution of the set concentration, (7) pumps, (8) conductometer, (9) immersion conductometric cell, (10–13) connecting hoses, (14) pH meter, and (15) combined glass electrode for pH measurements; Figure S5: Schematic design of the experimental setup (a) and plexiglass frames with special comb-shaped guides that separate the membranes (b): a flow-through four-compartment electrodialysis cell containing an anion-exchange membrane under study (AEM*) and two auxiliary membranes, an anion-exchange and a cation-exchange membranes; tank with 0.02 M electrolyte solutions (1); additional tank (2) for determination of ion transport numbers; valves (3, 4); the Luggin capillaries (5); Ag/AgCl electrodes (6); platinum polarizing the working and counter electrodes (7); Autolab PGSTAT100N (8); flow-through cell with a pH combination electrode (9); pH meter pHM120 MeterLab (10) connected to computer; pH meter (10); combined electrode for pH measurements (11) connected to pH meter (10); conductivity cell (12) connected to a conductometer; titration device (13) for maintaining a constant pH in the solution circulating through tank (2); desalination compartment (14); the solid purple lines show schematic concentration profiles in two neighboring compartments separated by the membrane under study; Figure S6: Concentration dependences of the CJMA-2 and the CJMA-2m membranes conductivity in Na_x_H_(3-x)_PO_4_ solutions with pH 4.5 ± 0.1 (a) and pH 9.5 ± 0.1 (b); Figure S7: Current-voltage curves of the studied membranes in 0.02 M NaCl solution (a), and the difference between the pH of the solution at the outlet and at the inlet of the desalination compartment (b) vs. the current density. The current density is normalized to the limiting current density calculated using the Leveque equation; Figure S8: Current-voltage curves of ASE, CJMA-3 membranes in 0.02 M Na_x_H_(3-x)_PO_4_ solutions with pH 4.4 ± 0.1 (a), 6.6 ± 0.1 (b), and 10.0 ± 0.2 (c). The current density is normalized to the limiting current calculated using the modified Leveque equation. Table S1: The values of pK_a_ (at 25 °C) of the orthophosphoric acid various species, which may be present in the membrane systems under study; Table S2: The rate constants of proton-transfer reactions (5)–(10) for the weak acids under study; Table S3: Some of the characteristics of the studied electrolytes, which are used to calculate the limiting currents; Table S4: The found values of the Leveque limiting current densities for 0.02 M *Na_x_H_(3-x)_PO_4_* solutions under study; Table S5: Some characteristics of the studied membranes. References [96,97,98,99,100,101,102,103,104,105,106,107,108,109,110,111,112,113,114,115,116,117,118,119,120,121,122,123,124,125,126,127,128,129,130,131,132,133,134] are cited in the Supplementary Materials.
