# Supplementary material for: Phosphates Transfer in Pristine and Modified CJMA-2 Membrane during Electrodialysis Processing of NaxH(3−x)PO4 Solutions with pH from 4.5 to 9.9
Source: Membranes (Basel). 2023 Jul 5;13(7):647. doi: 10.3390/membranes13070647 (PMC10386648; doi:10.3390/membranes13070647)
Supplement: Supplementary file 1 [file membranes-13-00647-s001.zip › membranes-2439293-supplementary.pdf]

# Phosphates transfer in pristine and modified CJMA-2 membrane during electrodialysis processing of $Na_xH_{(3-x)}PO_4$ solutions with pH from 4.5 to 9.9

**Natalia Pismenskaya, Olesya Rybalkina, Ksenia Solonchenko, Dmitrii Butylskii and Victor Nikonenko***

Kuban State University, 149, Stavropolskaya Str., Krasnodar, 350040, Russian Federation; n_pismen@mail.ru (N.P.); olesia93rus@mail.ru (O.R.); sol.ksenia17@yandex.ru (K.S.); dmitrybutylsky@mail.ru (D.B.); v_nikonenko@mail.ru (V.N.)
* Correspondence: v_nikonenko@mail.ru; Tel.: +7-918-41-45-816

## Supplementary materials

### *S1 Heterogeneous cation exchange (MC-40) and anion exchange (MA-41) membranes*

Heterogeneous cation exchange MC-40 membrane and anion exchange MA-41 membrane are made by hot rolling of milled cation-exchange resins KU-2-8 and anion –exchange resin AV-17-8, as well as a high-density polyethylene powder. Then reinforcing nylon mesh is introduced (Figure S1a) using the hot pressing method [1]. The size of ion-exchange resin particles ranges from 5 to 50 μm. The adhesion between individual resin particles, polyethylene, and the reinforcing cloth is low, resulting in gaps that form macropores (about 1 μm in size) when the membrane contacts solutions [2]. The resin particles are evenly distributed throughout the IEM (Figure S1b); their tops extend beyond the polyethylene-coated surface of heterogeneous membranes (Figure S1b). The resin is a copolymer of styrene and divinylbenzene (8%). It contains sulphonate fixed groups (KU-2-8) or quaternary ammonium bases as fixed functional groups and small amounts of weakly basic primary and secondary amines (AV-17-8).

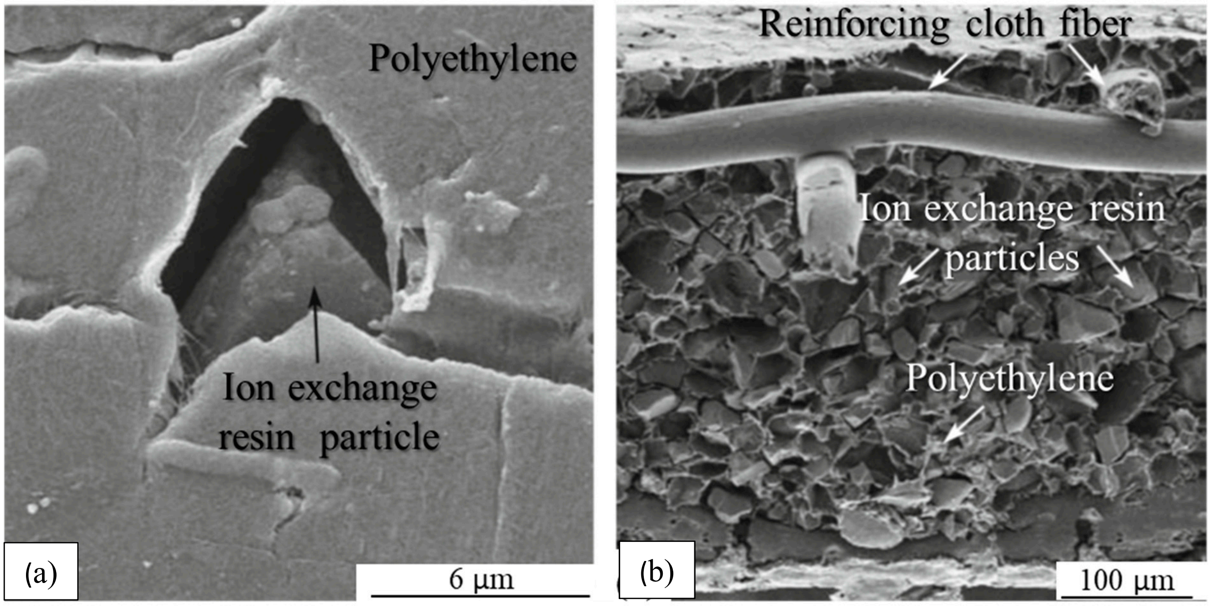


**Figure S1.** SEM images of (a) surfaces and cross-section of the heterogeneous MC-40 membrane (b). The heterogeneous membrane MA-41 has a structure similar to that of MC-40.

***S2 Solutions***

$Na_xH_{(x-3)}PO_4$ are the salts of the tribasic orthophosphoric acid. The latter has the following structure

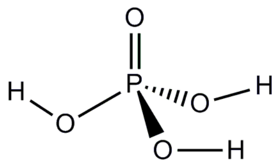

The proton-transfer reactions between water molecules and orthophosphoric acid species (the case of orthophosphoric acid with general formula $H_nA$, where the maximum value of n is 3) are presented as follows:

$$H_2A^- + H_2O \underset{k_{-1}}{\overset{k_1}{\rightleftarrows}} H_3A + OH^- ; \qquad K_{b1} = \frac{c_{H_3A} c_{OH^-}}{c_{H_2A^-}} \tag{S1}$$

$$H_3A + H_2O \underset{k_{-2}}{\overset{k_2}{\rightleftarrows}} H_2A^- + H_3O^+ ; \qquad K_{a1} = \frac{c_{H_2A^-} c_{H^+}}{c_{H_3A}} \tag{S2}$$

$$HA^{2-} + H_2O \underset{k_{-3}}{\overset{k_3}{\rightleftarrows}} H_2A^- + OH^- ; \qquad K_{b2} = \frac{c_{H_2A^-} c_{OH^-}}{c_{HA^{2-}}} \tag{S3}$$

$$H_2A^- + H_2O \underset{k_{-4}}{\overset{k_4}{\rightleftarrows}} HA^{2-} + H_3O^+ ; \qquad K_{a2} = \frac{c_{HA^{2-}} c_{H^+}}{c_{H_2A^-}} \tag{S4}$$

$$A^{3-} + H_2O \underset{k_{-5}}{\overset{k_5}{\rightleftarrows}} HA^{2-} + OH^- ; \qquad K_{b3} = \frac{c_{HA^{2-}} c_{OH^-}}{c_{A^{3-}}} \tag{S5}$$

$$HA^{2-} + H_2O \underset{k_{-6}}{\overset{k_6}{\rightleftarrows}} A^{3-} + H_3O^+ ; \qquad K_{a3} = \frac{c_{A^{3-}} c_{H^+}}{c_{HA^{2-}}} \tag{S6}$$

Figure S2. shows the distribution of species of the polybasic acids under study (in mole fractions) vs. the pH of the solution. This distribution is calculated using the appropriate equilibrium equations and the $pK_a$ values presented in Table S1.

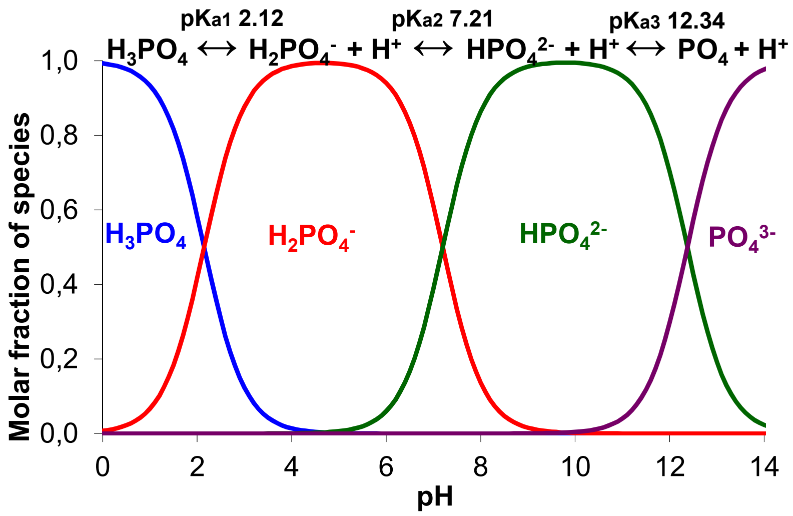


**Figure S2.** The distribution of species of the orthophosphoric acid (in mole fractions) vs. the pH of the solution.

### Calculation of the rate constants

There are relationships between the pseudo-unimolecular forward rate constants and backward rate constants, which involve the equilibrium constants, the acid dissociation ($K_a$) or the base ionization ($K_b$) constants. For example, in the case of reactions (S1) and (S2), we have[3,4]:

$$\frac{k_1'}{k_{-1}} = K_{b1} = \frac{K_w}{K_{a1}}; \qquad \frac{k_2'}{k_{-2}} = K_{a1} = \frac{K_w}{K_{b1}} \qquad \text{(S7)}$$

where $K_w$ (equal to $10^{-14}$ mol$^2$ dm$^{-6}$) is the water dissociation constant; $K_{ai}$ (mol dm$^{-3}$) is defined for each step of dissociation $i$=1, 2, 3 by Eqs. (S2), (S4) and (S6), respectively; $K_{bi} = K_w / K_{ai}$. Equations, similar to Eqs. (S7), can also be written for the 2-nd and 3-rd dissociation steps. The pseudo-unimolecular forward rate constants $k_1'$ and $k_2'$ are obtained, when the concentration of water, $c_{H_2O}$, is considered as a constant whose value is taken into account in the value of these constants: $k_1' = k_1 c_{H_2O}$, $k_2' = k_2 c_{H_2O}$ [3,5].

The values of the dissociation constants, $K_{ai}$, used in the calculations are given in Table S1. The values of the backward rate constants $k_{-1}$ and $k_{-2}$ are both taken equal to $10^{10}$ dm$^{-3}$ mol$^{-1}$ s$^{-1}$[3].

### Table S1

The values of p$K_a$ (at 25 °C) of the orthophosphoric acid [6] various species, which may be present in the membrane systems under study.

| Substance | p$K_{a1}$ | p$K_{a2}$ | p$K_{a3}$ |
|---|---|---|---|
| $H_3PO_4$ | 2.12 | 7.21 | 12.34 |

**Table S2**

The rate constants of proton-transfer reactions (5)-(10) for the weak acids under study.

| | 1-st step | | 2-nd step | | 3-rd step | |
|---|---|---|---|---|---|---|
| reactions, Eqs. | (7) | (8) | (9) | (10) | (11) | (12) |
| | $k'_1$, s$^{-1}$ | $k'_2$, s$^{-1}$ | $k'_3$, s$^{-1}$ | $k'_4$, s$^{-1}$ | $k'_5$, s$^{-1}$ | $k'_6$, s$^{-1}$ |
| $H_3PO_4$ | $1\times10^{-2}$ | $8\times10^{7}$ | $2\times10^{3}$ | $6\times10^{2}$ | $2\times10^{8}$ | $5\times10^{-3}$ |

## *S3 Methods*

***FTIR spectra*** of the membranes were obtained using a Vertex-70 spectrometer (Bruker Optics, Ettlingen, Germany) and OPUS™ 7.5 software.

***Contact Angles Measurements.*** The contact angles (θ) of the membranes under study were measured using the sessile drop technique [7]. The studied membrane was in a swollen state, it was removed from the $H_2O$ solution immediately before the measurements. The contact angles reported in this paper were registered 20 s after the applying a drop of distilled water on the membrane surface.

**The total exchange capacity ($Q_{sw}$)** of studied CEM under study is determined by the static method [8]. A sample of the swollen membrane (about 1.0 g (msw)), was initially transformed into the $H^+$ form by soaking in a 1M HCl solution. Then, after careful rinsing in deionized water, the sample was immersed in a 20.0 cm$^3$ of a 0.1M NaCl solution to replace $H^+$ ions by $Na^+$; it was kept there until equilibrium (for 24 hours). After that, the concentration of the $H^+$ ions released into the solution was determined using the potentiometric titration with a 0.1 M NaOH. The titration was performed using EasyPlusTitrators (METTLER TOLEDO), with the output of the titration results to a computer.

The calculation of the membrane exchange capacity per weight of swollen membrane, $Q_{sw}$ (mmol gsw$^{-1}$), was carried out by equation:

$$Q_{sw} = \frac{V_T c_T}{m_{sw}} \qquad \text{(S8)}$$

where VT is the volume of 0.1 M NaOH solution, spent on titration (mL), $c_T$= 0.1 mmol/mL (NaOH); $m_{sw}$ is the mass of swollen sample (g).

***Water content*** (W, %) of the membranes was determined by the gravimetric method. Before the experiment, the samples were equilibrated with a 0.02 eq L$^{-1}$ NaCl solution at 25 ± 1 °C for 24 h. After equilibration, samples were taken out from the solution and the film of liquid was removed from the samples' ends and surfaces using filter paper.

Weights of wet, $m_{wet}$, and dry, $m_{dry}$, samples were obtained using an MB25 Ohaus moisture analyzer. The evaporation of water was carried out at a temperature of 50 °C to a constant weight of the sample.

The water content W, % was calculated by the formula:

$$W = \frac{m_{wet} - m_{dry}}{m_{dry}} \times 100\% \qquad (S9)$$

***Electrical conductivity of anion–exchange membranes*** (κ*) was determined by a differential method using a clip cell [9] and a AKIP 6104 immitance meter (Motech Industries Inc., Taiwan) at an AC frequency of 1 kHz. All samples were studied in 0.02–1.0 eq $L^{-1}$ solutions of NaCl and $NaH_2PO_4$ (pH 4.4±0.1) starting from the lowest concentration.

The conductivity of the membranes (κ*) was found by the formula:

$$\kappa^* = \frac{d_m}{R_{m+s} - R_s} \qquad (S10)$$

where $R_{m+s}$ is the resistance of the membrane in solution and $R_s$ is the resistance of the solution alone.

According to the microheterogeneous model, an ion exchange membrane (IEM) is considered as a two-phase system involving a gel phase and intergel spaces filed with an equilibrium electroneutral solution. The volume fractions of these phases are $f_1$ and $f_2$, $(f_1 + f_2 = 1)$.

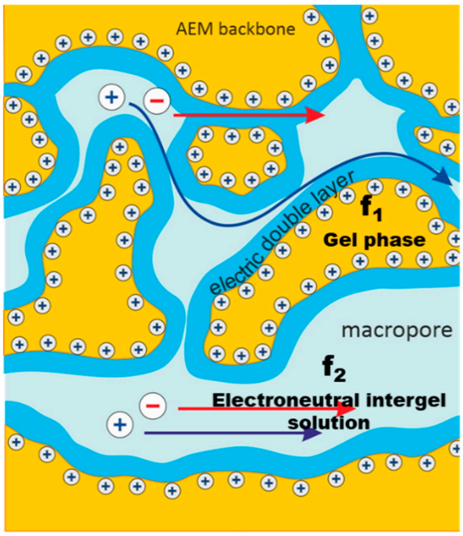


**Figure S3.** Cross-section of the ion exchange membrane volume in the framework of microheterogeneous model [10].

The gel phase is a microporous swollen medium (Figure S3). It includes the polymer matrix, which bears charged fixed groups, and the charged solution of mobile counterions and, in a smaller number, coions that compensate for the charge of fixed groups. The reinforcing cloth fibers and the inert filler (polyethylene) are also included in the gel phase. The second phase

includes the solution in the central part of the meso- and macropores and in the structural defects of the membrane.

In the first approximation (when the presence of coions in the gel phase is neglected), the electrical conductivity of this phase is considered to be constant, depending on the counterion diffusion coefficient in the gel phase of the membrane, $\overline{D_i}$, and on its exchange capacity, $\bar{Q}$:

$$\bar{\kappa} = \frac{z_i \bar{D}_i \bar{Q} F^2}{RT}, \quad \text{(S11)}$$

where $F$ is the Faraday constant, $R$ is the universal gas constant, $T$ is the temperature, and $z_i$ is the counterion charge. The value is related to the ion-exchange capacity $\bar{Q}$ of the membrane by $\bar{Q} = Q/f_1$. The numerical value of $\bar{\kappa}$ can be determined from the value of the membrane electrical conductivity ($\kappa^*$) at the isoconductivity point in which the conductivities of the membrane ($\kappa^*$) and the solution ($\kappa$) are identical. It is clear that according to Equation (S17), the following equality holds in this case: $\kappa^*= \kappa =\bar{\kappa}$. For commercial membranes and strong electrolytes, the concentration at this point ($C_{iso}$,) is not far from 0.05 mol L$^{-1}$ solution: at $C > C_{iso}$, the conductivity of the membrane is higher than that of the solution, $\kappa^* > \kappa$, and at $C > C_{iso}$, $\kappa^* < \kappa$.

According to the microheterogeneous model [11], within the concentration range $0.1c_{iso} < c < 10c_{iso}$, and under condition that parameter α is not too great ($|\alpha| \leq 0.2$), the electrical conductivity of an IEM, $\kappa^*$, can be expressed as:

$$\kappa^* = \bar{\kappa}^{f_1}\kappa^{f_2} \quad \text{(S12)}$$

where the conductivity of the “intergel” solution, $\kappa$, is assumed to be equal to that of the external equilibrium solution.

***The diffusion characteristics of IEMs*** were studied using two-compartment flow-through cell *1* [12] equipped with special devices for the inlet and outlet of the solution (Figure S4). These devices provide the laminar flow of the solution in cell compartments *3* and *4*, which makes it possible to use the Leveque equation for calculating the average thickness of diffusion layers, δ, near the surface of the IEM under study [13]:

$$\delta = 1.02(LDh/\bar{V})^{1/3} \quad \text{(S13)}$$

where $L$ is the length of the channel, cm; $h$ is the distance between test membrane *2* and the cell wall, cm; $D$ is the diffusion coefficient of the electrolyte, cm$^2$/s; and $\overline{V}$ is the average linear flow velocity of the solution, cm s$^{-1}$. The values of $h$ were 0.63 cm, and the working area of the membranes $S$ was 2.7×2.7 cm$^2$.

The membrane under study, which was preliminarily equilibrated with a 0.1 M electrolyte solution (NaCl, $NaH_2PO_4$) was placed into cell *1*. Prior to each experiment, 1000 cm$^3$ of the

electrolyte solution with a set concentration was poured into flowing tank *6* and compartment *4* (stream II). Distilled water in an amount of 100 $cm^3$ was poured into flowing tank *5* and compartment *3* (stream I). In 40 min after starting the experiment, the solution from this duct was rapidly drained, and the tank was refilled with distilled water. The average linear velocity of the solutions circulating through each of the cell compartments was 0.90±0.10 cm $s^{-1}$. Every 30 s, the values of the electric conductivity and pH of the solution in container *5* were recorded using immersion conductivity cell *9* and combined glass electrode *15* connected to an Expert 001 conductometer and an Expert 002 pH meter.

The values of the integral diffusion permeability coefficient were calculated according to the known equation [14]

$$P = \frac{d_m V_I}{S C_{II}} \frac{dC_I}{dt}, \tag{S14}$$

where $d_m$ is the thickness, $S$ is the area of the membrane under study, $V_I$ is the volume of the solution in tank I, $C_{II}$ is the concentration of the solution in tank II, and $dC_I/dt$ is the rate of concentration growth in stream I. The latter is determined using the regression analysis of the linear portion of the time-dependence curve of the electric conductivity of the solution in container *5* (initially filled with pure water). The studies were performed at different concentrations of NaCl in stream II.

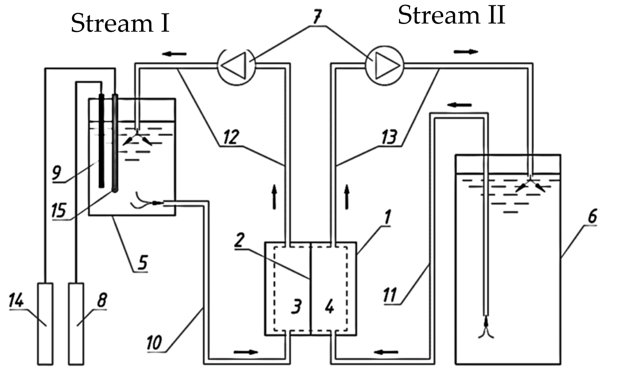


**Figure S4**. Schematic of the unit for measuring the diffusion permeability of membranes: (1) two-compartment cell, (2) membrane under study, (3, 4) flow-through compartments of cell 1, (5) tank with distilled water, (6) tank with an electrolyte solution of the set concentration, (7) pumps, (8) conductometer, (9) immersion conductometric cell, (10–13) connecting hoses, (14) pH meter, and (15) combined glass electrode for pH measurements.

$P$ is a characteristic convenient for the practical use. However, in theoretical considerations, the differential (or local) diffusion permeability coefficient, $P*$, is often applied. The relation between these characteristics is given as $P = \frac{1}{C}\int_0^C P^* dC$, which leads to the following formula:

$$P^* = P + C\frac{dP}{dC} \tag{S15}$$

In practice, for calculation $P*$, it is more convenient to use the relationship between $P*$ and $P$ in the form:

$$P^* = P(\beta + 1), \quad \text{(S16)}$$

with $\beta$=*dlgP/dlgC*, which is the slope of the *lgP vs lgC* dependence.

Knowing the values of $\kappa^*$ and $P^*$, it is possible to find the transport numbers of counterions ($t_1^*$) and coions ($t_A^*$) in a membrane:

$$t_1^* = \frac{1}{2} + \sqrt{\frac{1}{4} - \frac{(z_1|z_A|)P^*F^2C}{(z_1+|z_A|)RT\kappa^*}}, \; t_A^* = 1 - t_1^* \quad \text{(S17)}$$

Here $z_1$ and $z_A$ are the charge numbers of the counterion and coion, respectively; *F* is the Faraday constant; *R* is the gas constant, *T* is the temperature.

***The current-voltage characteristics** (CVC) **and the pH difference between the outlet and inlet solutions of the desalination compartment.*** CVCs were recorded at the current sweep of 0.02 mA $s^{-1}$. The working area of measuring Ag/AgCl electrodes immersed in the solution identical to the feed solution is 11.2 $cm^2$. In the case of phosphate, tartrate or citrate containing solution, a small amount of NaCl (~1%) was added to the solution to ensure the stable operation of the measuring electrodes. The initial volume (before the experiment) of the feed solution in tank (1) and the hoses was 5 L. The feed solution was pumped from tank (1) through all the cell compartments; then, it returned into the same tank. The salt concentration in the feed solution circulating through tank (1) changed very little (<1%) during one experimental run due to the relatively large volume of this solution.

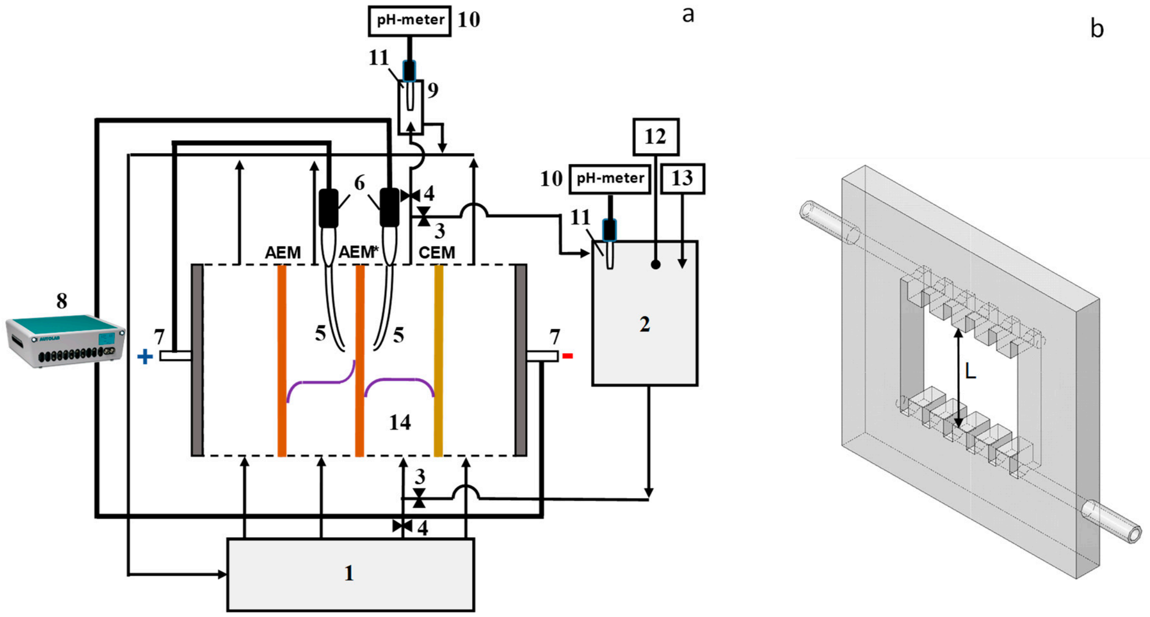


**Figure S5.** Schematic design of the experimental setup (a) and plexiglass frames with special comb-shaped guides that separate the membranes (b): a flow-through four-compartment electrodialysis cell containing an anion-exchange membrane under study (AEM*) and two auxiliary membranes, an anion-exchange and a cation-exchange membranes; tank with 0.02 M electrolyte solutions (1); additional tank (2) for determination of ion transport numbers; valves (3, 4); the Luggin capillaries (5); Ag/AgCl electrodes (6); platinum polarizing the working and counter electrodes (7); Autolab PGSTAT100N (8); flow-through cell with a pH combination electrode (9); pH meter pHM120 MeterLab (10) connected to computer; pH meter (10); combined electrode for pH measurements (11) connected to pH meter (10);

conductivity cell (12) connected to a conductometer; titration device (13) for maintaining a constant pH in the solution circulating through tank (2); desalination compartment (14); the solid purple lines show schematic concentration profiles in two neighboring compartments separated by the membrane under study.

The potential drop over the membrane under study (AEM*) measured using Luggin's capillaries (5) is a function of the distance between the capillary tip and the membrane as well as the ohmic resistance produced by the membrane [15]. When the cell is disassembled for replacing the membrane, these parameters change. To exclude this ambiguity, instead of the total potential drop, $\Delta\varphi$, we use the corrected potential drop, $\Delta\varphi'$, defined as follows [16]:

$$\Delta\varphi' = \Delta\varphi - iR_{ef} \quad \text{(S18)}$$

where $R_{ef}$ (Ohm cm$^2$) is the effective resistance, which is found by extrapolation $i \rightarrow 0$ in the coordinates $i - d\varphi / di$, using the initial part of CVC [9].

*Electrochemical impedance spectra (EIS)* are obtained using the same cell / set-up as in the case of CVC measurement. In the case of CVC measurement, closed measuring Ag/AgCl electrodes of the EVL-1M3.1 brand with the working area of several tenths of cm2 immersed in the saturated KCl solution were used. In the case of impedancemetry, open Ag/AgCl electrodes with working area of 11.2 cm2 immersed in the same solution as used in the ED cell were applied. The time required for obtaining each spectrum was about 2 hours. First, the membrane was maintained for 20 min at a given direct current (DC) density i (in the range 0 from to 0.018mA) then an alternating current (AC) equal to 0.25 mA was applied over the direct current. The membrane was kept for several minutes at a given frequency until reaching a stationary state (its achievement was determined automatically by the Autolab PGSTAT-100 complex). The measurements were carried out in the frequency range from 3×10-3 Hz to 5×105 Hz, starting from the lowest frequency. EIS were obtained at different values of DC density, starting from the smallest current. The interval between two consecutive measurements, during which i = 0, was 40 minutes.

Three main elements can be distinguished in the presented spectra: the high-frequency element (the corresponding arc of a semi-circle is indicated by index I), the low-frequency arc of finite-length Warburg impedance (indicated by index II) and the arc of Gerischer impedance (indicated by index III). The latter is recorded at medium frequencies and appears when a chemical reaction takes place at the membrane / solution interface. According to modern concepts [17–20], the high-frequency arc is determined by the geometric capacity and / or capacity of the double layer at the membrane / solution interface, $C_I$ (measured in $F_1=C_1/V_1$), as well as the ohmic resistance of the system under study (generally polarized by DC) (in Ohm=V/A) [18,21,22].

The characteristic frequency $f_I$ (in $s^{-1}$), which corresponds to the top of this arc, is determined by the equation [23]:

$$f_I = \frac{1}{2\pi C_I R_I} \qquad \text{(S19)}$$

In the Nyquist coordinates, $R_I$ is equal to the diameter of the first high-frequency arc. This resistance is the response of the system under study to such rapid changes in the alternating current that the concentration of the electrolyte in the solution does not have time to change. $R_I$ depends on the DC density causing the concentration polarization of the system. The concentration polarization leads to changes in the resistance of the diffusion layers adjacent to the membrane, primarily, in the depleted diffusion layer. Theoretical estimates of the characteristic frequency $f_I$, which corresponds to the maximum point on the high-frequency arc, give a value of about 100 kHz [17,18].

The occurrence of the Warburg impedance arc is due to the transformation of concentration changes in the quasi-electroneutral solution adjacent to the membrane into changes in the potential drop, $\Delta\varphi$. If the current varies, the changes of the concentration occur with a delay in time due to the diffusion of the solute. Accordingly, changes in $\Delta\varphi$ in response to variation in the current also occur with a delay [17,24,25].

The time scale of diffusion processes depends on the characteristic length and mobility of ions in specific zones of the membrane system: diffusion boundary layer, membrane, electric double layer [17,26]. Generally, the depleted diffusion boundary layer in the solution (of thickness δ) plays a dominant role in the formation of the Warburg impedance. In this case, the characteristic frequency is determined by the expression [27]

$$f_{II} = \frac{2.54}{2\pi}\frac{D}{\delta^2} \qquad \text{(S20)}$$

The Gerischer impedance is characterized by the effective resistance of the chemical reaction, $R_G$, which occurs at the membrane / solution interface. The value of $R_G$ is equal to the diameter of the Gerischer arc. The frequency related to the maximum point of this arc, $f_{III}$, is connected with the effective rate constant of the water splitting reaction, $\chi$, by the equation [28]

$$f_{III} = \frac{\chi\sqrt{3}}{2\pi} \qquad \text{(S21)}$$

Thus, the alternating electric current in a membrane system causes several processes. First of all, it is the capacitive polarization of the membrane system, which occurs due to the presence of the geometrical capacitance of the diffusion layers and the capacitance of the electrical

double layer. This process is described by the first high-frequency arc. In addition, there are diffusion in the solution adjacent to the membrane (the finite-length Warburg arc) and a chemical reaction (water splitting) at the membrane / solution interface (the Gerischer arc). The greater the chord length of the arc, the higher the contribution of the corresponding process to the behavior of the system. Each process has its own frequency range. However, if the arc relating to a process is small, it can be absorbed by the neighboring arc of another dominant process.

***Electrodialysis processing of the phosphate solution*** was carried out using the cell presented in Figure S5. The difference with the measurements of CVC is in the fact that desalination compartment (14) is fed from additional tank (2). The volume of the solution circulating through the desalination compartment and tank (2) is 0.1 L, which is essentially less than the volume of the solution circulating through tank (1), the concentration and electrode compartments. During one experimental run, in conditions where the current density in the electrodialysis cell potential is kept constant, the salt concentration in the diluate stream decreased with time. Since the rates of generation of $H^+$ and $OH^-$ ions at the CEM and AEM forming the desalination compartment are different, pH of the feed solution changed with time. Namely, it became acidic in the studied cases. In order to keep a constant pH value of the feed solution, a 0.1M solution of NaOH was added into tank (2) through microcapillary (13). The rate of decrease in the salt concentration of the solution in tank (2), *dC/dt*, is found by using the measured values of conductivity, $\kappa$, of this solution (using submersible conductometric cell (12)) and taking into account the known constant pH value. The potential drop was measured during the entire experiment with an interval of 300 s. The measurements were carried out using Luggin capillaries (5), located near the center of the polarizable surfaces of the membrane under study. The distance between the membrane surface and the tip of the capillary was about 0.8 mm.

The desalination degree, $\boldsymbol{\gamma_D}$ , and the component *i* recovery degree, $\boldsymbol{\gamma_i}$ , were found by the equations:

$$\gamma_D = \frac{\kappa^0 - \kappa^t}{\kappa^0} \tag{S22}$$

$$\gamma_i = \frac{c_i^0 - c_i^t}{c_i^0} \tag{S23}$$

where the indices «0» and «t» correspond to the characteristics of the diluate stream at the beginning and after a given time of electrodialysis.

Electric charge, *Q*, and energy consumption, *W*, spent on the desalination of the solution was determined as:

$$\mathrm{Q} = \mathrm{I_{av}t} \tag{S24}$$

$$W=\int I(t)\Delta\varphi(t)dt \quad \text{(S25)}$$

where $I_{av}$ – is the average current intensity over the period. The value of $W$ was calculated using equation (S26), according to the dependences of $I(t)$ and $\Delta\varphi(t)$ measured using the Autolab PGSTAT100 electrochemical complex. Integration in accordance with equation (S15) was carried out from the beginning of the ED process, $t$=0, to the time $t=t'$, when the desired desalination degree was reached (50%).

The current efficiency were calculated using the equation:

$$\eta = \frac{z_i F \bar{V}(C_{0i} - C_{ti})}{n\int_0^t I_{tot}(t)\,dt} \quad \text{(S26)}$$

## *S4 Theoretical limiting current calculations*

*The Lévêque limiting current density in NaCl solution*

The Leveque equation [(S27) and equation (S28)] are used to estimate the theoretical limiting current, $i_{lim}^{Lev}$, and the thickness of the depleted diffusion layer, $\delta^{Lev}$ in NaCl solution:

$$i_{\lim}^{Lev} = \frac{z_1 F D c_1^0}{h(T_1 - t_1)}\left[1.47\left(\frac{h^2 V_0}{LD}\right)^{1/3}\right] \quad \text{(S27)}$$

$$\delta^{Lev} = 0.71h\left(\frac{LD}{h^2 V_0}\right)^{1/3} \quad \text{(S28)}$$

Here $z_1$ is the charge number of counterion 1, $F$ is Faraday constant, $D$ и $t_1$ are the diffusion coefficient of the electrolyte and the electromigration transfer number of the counterion at infinite dilution of the solution. The transport number of counterions in the membrane, $T_1$, was considered equal to one; $c_1$ is its molar concentration in the feed solution entering the DC, $V_0$ is the average linear velocity of the solution flowing between the membranes forming the DC, $h$ is the distance between the membranes, $L$ is the length of the channel. These equations were obtained for the diffusion-convection heat transfer [29] and after were adapted to diffusion-convection mass transfer in electrode [30] and ion exchange membrane [31] systems. For 1: 1 electrolyte and laminar hydrodynamic regime. Note that the value 0.71 for the factor in the right-hand part of Eq. (S28). However, often [32,33] Eq. (S28) is used with a factor 0.68, which is obtained using the Peers equation [34] and the approximation of Eq. (S27), where the second term in the brackets is neglected. It is worth noting that this equation is applicable only for relatively short channel lengths ($L \le 0.02h^2V_0 / D$) [8].

*The Lévêque limiting current density in a mixed electrolyte solution ($Na_xH_{(3-x)}PO_4$)*

Let us consider a ternary electrolyte composed of two kinds of counterions, 1 and 2, and one kind of coion, a later on, we consider a solution of this ternary electrolyte in a diffusion layer adjacent to an ion-exchange membrane.

The Nernst-Planck equations for these ions read:

$$j_1 = -D_1\left(\frac{dc_1}{dx} + z_1 c_1 \frac{F}{RT}\frac{d\varphi}{dx}\right) \tag{S29}$$

$$j_2 = -D_2\left(\frac{dc_2}{dx} + z_2 c_2 \frac{F}{RT}\frac{d\varphi}{dx}\right) \tag{S30}$$

$$j_A = -D_A\left(\frac{dc_A}{dx} + z_A c_A \frac{F}{RT}\frac{d\varphi}{dx}\right) \tag{S31}$$

After dividing each of the equations (S29)-(S31) by $D_i$, summing the results, and taking into account the electroneutrality condition

$$z_1c_1 + z_2c_2 + z_Ac_A = 0 \tag{S32}$$

we find:

$$\frac{j_1}{D_1} + \frac{j_2}{D_2} + \frac{j_A}{D_A} = -\frac{d\left(c_1 + c_2 + c_3\right)}{dx} = -\frac{\left(1+\left|z_1/z_A\right|\right)dc_1}{dx} - \frac{\left(1+\left|z_2/z_A\right|\right)dc_2}{dx} \tag{S33}$$

The last equality in Eq. (S33) is obtained after eliminating $c_A$ using Eq. (S32). Since $j_i$ do not change along the coordinate $x$ in a stationary state, Eq. (S33) can be easily integrated over the thickness of the diffusion layer. If the current density is equal to the limiting one, the concentrations of all ions at the membrane surface are very close to zero. In this case, we can write:

$$\frac{j_{1\lim}^{theor}}{D_1} + \frac{j_{2\lim}^{theor}}{D_2} = \frac{\left(1+\left|z_1/z_A\right|\right)c_1^0}{\delta} + \frac{\left(1+\left|z_2/z_A\right|\right)c_2^0}{\delta} - \frac{j_{A\lim}^{theor}}{D_A} \tag{S34}$$

It follows from Eq. (S27) that the limiting flux density of counterion $i$ can be represented as [35]:

$$j_{i\lim}^{theor} = \frac{\left(1+\left|z_i/z_A\right|\right)D_ic_i^0}{\delta} - \frac{\left|z_i\right|D_ic_i^0}{\left|z_A\right|D_Ac_A^0}\cdot j_{A\lim}^{theor} \tag{S35}$$

The first term in Eq. (S36) shows what value the limiting flux density of counterion $i$ would have if the membrane were impermeable to coions. The second term reflects the additional value of the counterion flux caused by the transfer of coions: when the coions appear in the depleted layer, they create an additional electric field that attracts counterions from the solution. This effect is called exaltation in the literature [29,36].

From Eq. (S36), it is easy to obtain an expression for the limiting current density:

$$i_{\lim} = z_1 j_1 + z_2 j_2 + z_A j_A = i^0_{\lim} + \left(\frac{T_{A_{\lim}}}{t_A}\right) i_{\lim} \tag{S36}$$

where $t_A = \dfrac{D_A z_A^2 c_A^0}{\sum_{i=1,2,A} D_i z_i^2 c_i^0}$ is the coion transport number in the bulk solution, $T_{A_{\lim}} = \dfrac{z_A j_{A\lim}^{theor} F}{i_{\lim}}$ is the coion effective transport number in the membrane at $i = i_{\lim}$,

$$i^0_{\lim} = \frac{\left[\left(1+\left|z_1/z_A\right|\right) D_1 z_1 c_1^0 + \left(1+\left|z_2/z_A\right|\right) D_2 z_2 c_2^0\right] F}{\delta} \tag{S37}$$

is the limiting current density in the case of a membrane impermeable to coions. The term $\left(T_{A_{\lim}} / t_A\right) i_{\lim}$ in Eq. (S37) can be interpreted as the sum of the current carried by the coions $\left(T_A \cdot i_{\lim}\right)$, and the exaltation current of counterions. From Eq. (S37), we obtain:

$$i_{\lim} = \frac{i^0_{\lim} \cdot t_A}{t_A - T_A} \tag{S38}$$

The resulting Eq. (S38) generalizes the well-known Peers equation for a single electrolyte. Indeed, setting $c_2^0 = 0$ gives:

$$i_{\lim} = \frac{D \cdot z_1 \cdot c_1^0 \cdot F}{\left(T_1 - t_1\right)\delta} \tag{S39}$$

where:

$$D = \frac{D_1 D_A \left(z_1 + \left|z_A\right|\right)}{D_1 z_1 + D_A \left|z_A\right|} \tag{S40}$$

is the electrolyte diffusion coefficient.

For a mixture of two single electrolytes with a common coion (a ternary electrolyte), one can obtain equation (S41), which is similar to equation (S39) [37]:

$$i_{\lim} = \frac{D_{ter} \cdot \left|z_A\right| \cdot c_A^0 \cdot F}{\left(t_A - T_A\right)\delta} \tag{S41}$$

with $D_{ter}$, the effective diffusion coefficient of ternary electrolyte,

$$D_{ter} = \left[\left(1+\left|\frac{z_1}{z_A}\right|\right) D_1 N_1 + \left(1+\left|\frac{z_2}{z_A}\right|\right) D_2 N_2\right] \cdot t_A = \frac{\left[\left(z_1+\left|z_A\right|\right) D_1 z_1 c_1^0 + \left(z_2+\left|z_A\right|\right) D_2 z_2 c_2^0\right] D_A}{\sum_{i=1,2,A} D_i z_i^2 c_i^0} \tag{S42}$$

$N_i = \dfrac{z_i c_i^0}{z_A c_A^0}$ is the equivalent fraction of counterion $i$ in the bulk solution. It is easy to see that in the case, where the concentration of counterion 2 is zero, $c_2^0 = 0$, Eq. (S41) is reduced to Eq. (S42).

For a single electrolyte, the Leveque equation (Eq. (27)), allows calculating the limiting current density and diffusion layer thickness as functions of the (single) electrolyte diffusion coefficient, solution flow rate, distance between the membranes and membrane length. It can be assumed that this equation remains valid in the case of ternary electrolyte (e.g. $Na_2HPO_4$ + $NaH_2PO_4$) if the value determined by Eq. (S42) is used as the electrolyte diffusion coefficient.

$$\delta^{Lev} = 0.68h\left(\frac{LD}{h^2V_0}\right)^{1/3} \tag{S43}$$

Table S2 summarizes some of the characteristics of the studied electrolytes, which are used to calculate the limiting currents.

***Table S3***

Some of the characteristics of the studied electrolytes, which are used to calculate the limiting currents.

| Electrolyte | Diffusion coefficients at infinite dilution, $D_i \times 10^5$, cm$^2$ s$^{-1}$ | | | | Transport numbers at infinite dilution, $t_i$ | | |
|---|---|---|---|---|---|---|---|
| | cation | anion | | electrolyte | cation | anion | |
| | | singly charged | doubly charged | | | singly charged | doubly charged |
| NaCl | 1.334 [38] | 2.032 [38] | | 1.61 | 0.396 | 0.604 | |
| $NaH_2PO_4$ | | 0.959 [38] | | 1.12 | 0.581 | 0.419 | |
| $Na_2HPO_4$ | | - | 0.759 [38] | | | | 0.456 |

Calculations made using equation S33 give the following value $D_{ter}$: $1.12 \cdot 10^{-9}$ cm$^2$ s$^{-1}$ (pH 4.4 ± 0.1), $1.20 \cdot 10^{-9}$ cm$^2$ s$^{-1}$ (pH 6.6 ± 0.1) and $1.38 \cdot 10^{-9}$ cm$^2$ s$^{-1}$ (pH 10.0 ± 0.2). It is calculated under the assumption that $Na^+$ is the only coion.

***Table S4***

The found values of the Leveque limiting current densities for 0.02 M $Na_xH_{(3-x)}PO_4$ solutions under study

| pH | Molar concentration, M | | | | $i_{lim}^{Lev}$, mA cm$^{-2}$ |
|---|---|---|---|---|---|
| | $NaH_2PO_4$ | $Na_2HPO_4$ | $Na_3PO_4$ | NaCl | |
| 4.5 ± 0.1 | $1.99\ 10^{-2}$ | $3.98\ 10^{-5}$ | $5.31\ 10^{-13}$ | - | 1.64 |
| 6.6 ± 0.1 | $1.60\ 10^{-2}$ | $4.03\ 10^{-3}$ | $6.77\ 10^{-9}$ | - | 2.04 |
| 9.9 ± 0.2 | $3.95\ 10^{-5}$ | $1.99\ 10^{-2}$ | $6.66\ 10^{-5}$ | - | 3.58 |

| | | | | | |
|---|---|---|---|---|---|
| 5.6 ± 0.1 | - | - | - | 2.0 $10^{-2}$ | 3.07 |

### *S5 Concentration dependences of CJMA-2 and CJMA-2m membranes conductivity*

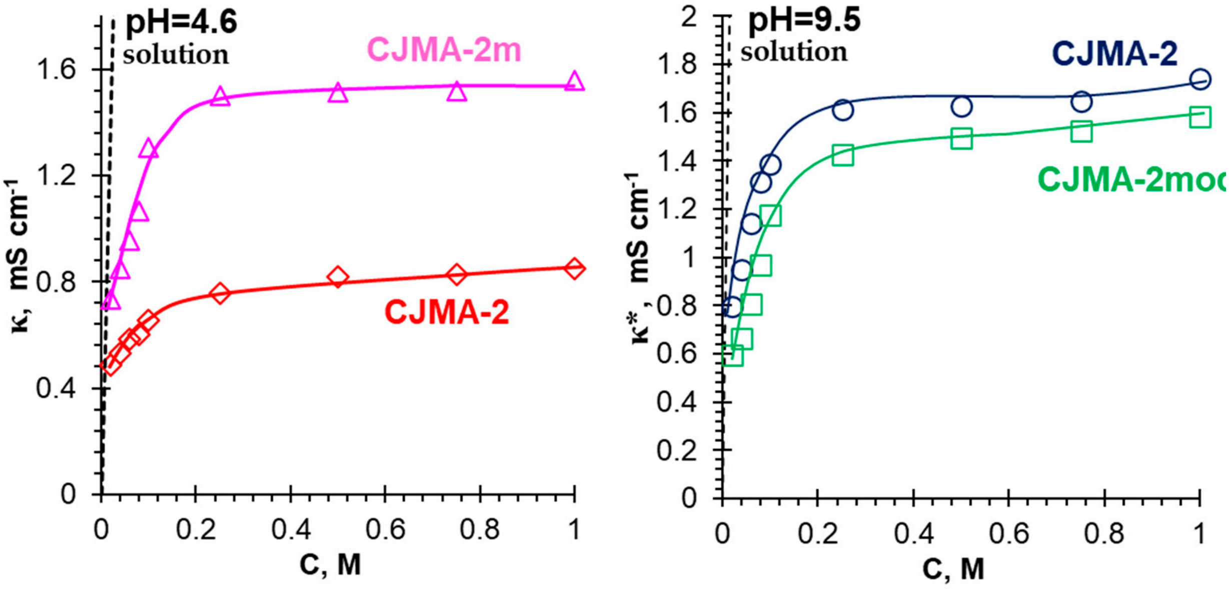


a b

**Figure S6.** Concentration dependences of the CJMA-2 and the CJMA-2m membranes conductivity in $Na_xH_{(3-x)}PO_4$ solutions with pH 4.5 ± 0.1 (a) and pH 9.5 ± 0.1 (b).

### *S6 Characteristics of some anion exchange membranes with quaternary ammonium groups*

Hefei Chemjoy Polymer Materials Co. Ltd. (Hefei, China) manufactures homogeneous anion exchange CJMA-3 and CJMA-6 membranes. The basis of their ion-exchange matrix are polyvinylidene fluoride (CJMA-3) [39] or polyolefins (CJMA-6). These matrixes are crosslinked with cross-linked agents [40]. Polyethylene terephthalate cloth provides mechanical strength of the membranes. Detailed information about their surface properties as well as diffusion permeability and selectivity is given in Refs. [41,42].

Astom (Yamaguchi, Japan), the manufacturer of the homogeneous Neosepta ASE anion-exchange membrane [43], does not provide information on its chemical structure. At the same time Chen et al. [44] report that this membrane has a polystyrene matrix cross-linked with divinylbenzene and contains strongly basic amino groups. The reinforcing cloth is made from a mixture of polyethylene and polypropylene.

***Table S5***

Some characteristics of the studied membranes

| Membrane | Ion-exchange capacity of wet membrane, mmol/$g_{wet}$ | Water Content, $gH_2O/g_{dry}$, % | Thickness in 0.02M NaCl solution, μm | $f_2$ in NaCl solution | $\overline{Q}$, mmol/$g_{wet}$ |
|---|---|---|---|---|---|

| | | | | | |
|---|---|---|---|---|---|
| CJMA-3 | 0.57 ± 0.05 [41] | 17 ± 1 [41] | 151 ± 5 | 0.27 ± 0.02 | 0.8 ± 0.1 |
| ASE | 1.93 ± 0.05 [45] | 20 ± 1 | 150 ± 5 | 0.06 ± 0.02 | 2.0 ± 0.1 |

### *S7 Current-voltage curves of anion-exchange membranes with quaternary ammonium groups in NaCl and $Na_xH_{(3-x)}PO_4$ solutions*

Current-voltage curves of ASE, CJMA-3 membranes in 0.02 M NaCl solution are shown in Figure S7a. Figure S7b presents the dependence of the difference between the pH of the solution at the outlet and at the inlet of the desalination compartment upon the current density, obtained simultaneously with the CVC.

The shape of these CVCs is typical for the curves obtained for membranes in solution of electrolytes that do not participate in protonation-deprotonation reactions [46,47].

Water splitting is minimal in the case of ASE due to the low catalytic activity of strong basic fixed groups towards water splitting [48,49].

The CJMA-3 membrane also contains predominantly strongly basic fixed groups. However, its surface with "valleys" and "hills" is very undulating [50]. A significant decrease in the concentration of NaCl in the stagnant zones of the "valleys" enhances water splitting (and acidification of the solution) compared to ASE.

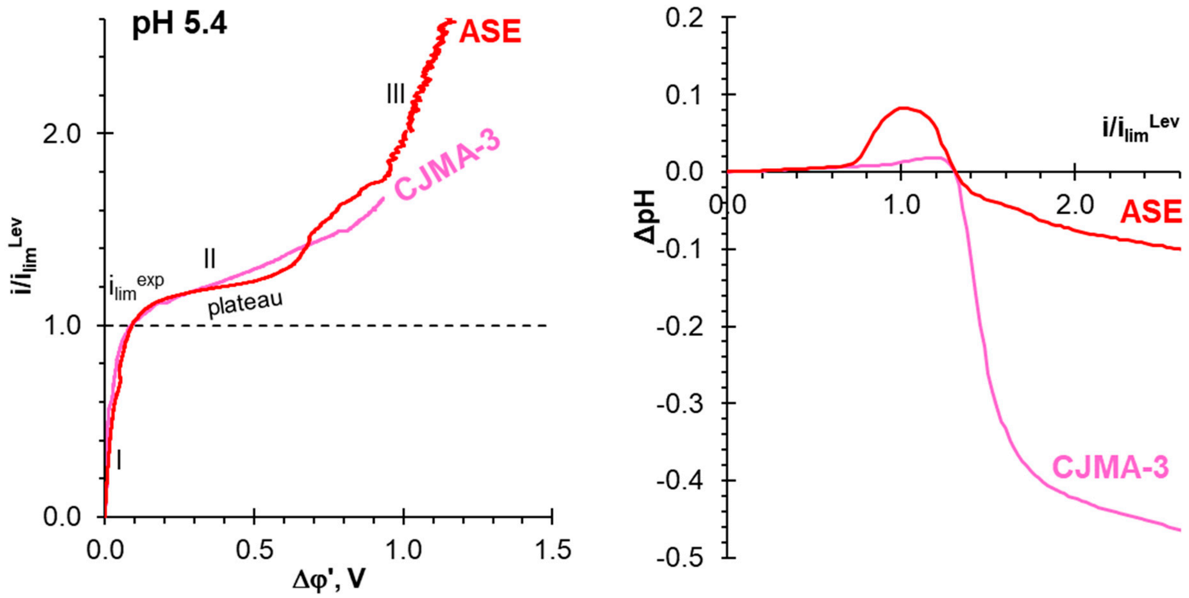


a b

**Figure S7.** Current-voltage curves of the studied membranes in 0.02 M NaCl solution (a), and the difference between the pH of the solution at the outlet and at the inlet of the desalination compartment (b) vs. the current density. The current density is normalized to the limiting current density calculated using the Leveque equation.

The occurrence of two sloping plateaus on the CVCs of ASE and CJMA-3 membranes (Figure S8a) is a consequence of the "acid dissociation" mechanism [51,52].

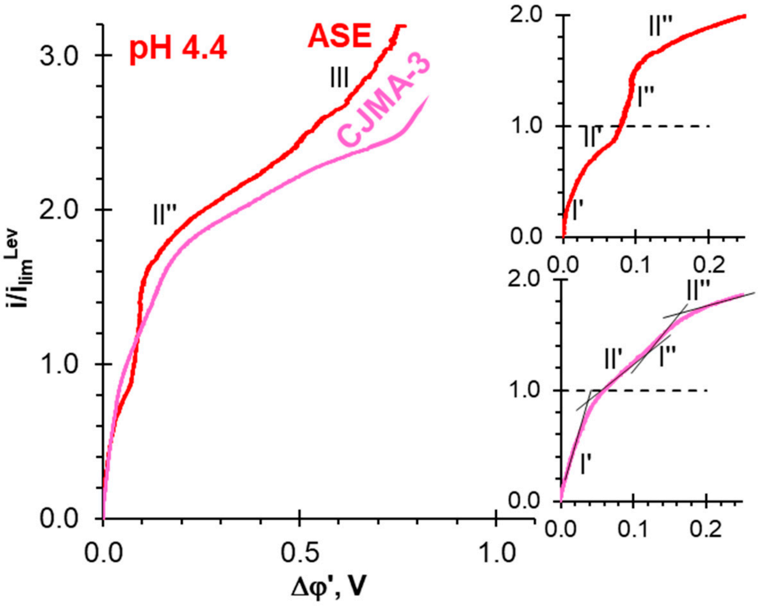


a

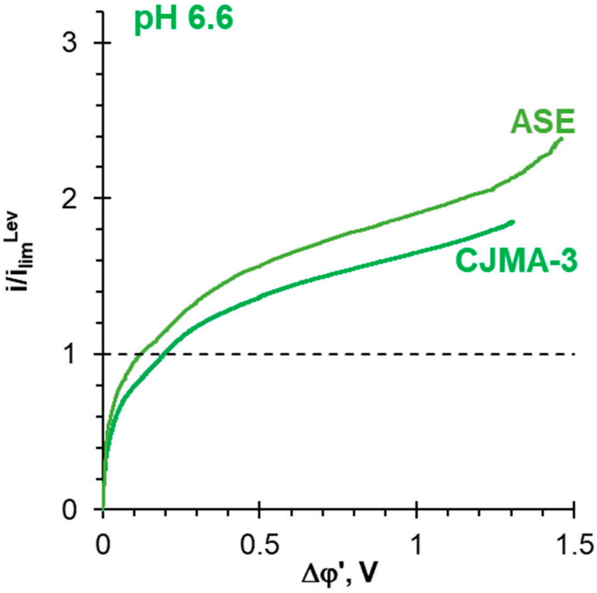


b

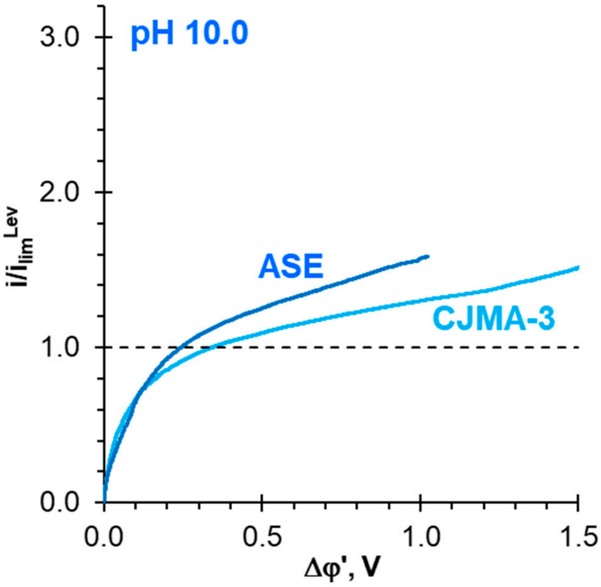


c

**Figure S8.** Current-voltage curves of ASE, CJMA-3 membranes in 0.02 M $Na_xH_{(3-x)}PO_4$ solutions with pH 4.4 ± 0.1 (a), 6.6 ± 0.1 (b), and 10.0 ± 0.2 (c). The current density is normalized to the limiting current calculated using the modified Leveque equation.

The ASE membrane has a higher ion-exchange capacity compared to the CJMA-3 membrane. This results in a stronger Donnan exclusion of protons in ASE compared to CJMA-3, due to a greater electrostatic repulsion force [53]. Accordingly, the value of the current density corresponding to plateau II" is higher in the case of ASE compared to CJMA-3.

$Na_xH_{(3-x)}PO_4$ solution with pH 10.0 ± 0.2 contains about 99% $H_2PO_4^-$ and 1% $PO_4^{3-}$. The pseudo-unimolecular rate constant of the rate-limiting step of the $HPO_4^{2-} \leftrightarrow PO_4^{3-} + H^+$ reaction is very low, $5 \cdot 10^{-3}$ $s^{-1}$ [52]. For the conversion of $H_2PO_4^-$ anions into $PO_4^{3-}$ anions due to the "acid dissociation" mechanism, a relatively high potential difference is needed.

Therefore, this mechanism has practically no effect on the shape of ASE and CJMA-3 current-voltage curves. This shape remains approximately the same as in the case of NaCl solutions. Acidification of the desalted solution at $i < i_{lim}^{Lev}$ does not exceed the experimental error (Figure S8b), but becomes more significant at $i > i_{lim}^{Lev}$.

The CVC of ASE and CJMA-3 membranes obtained in $Na_xH_{(3-x)}PO_4$ solution with pH 6.6 ± 0.1, which contains about 20% $HPO_4^{2-}$ and 80% $H_2PO_4^-$, have an intermediate shape (Figure 8b). Since this solution has buffer properties, "acid dissociation" and water splitting mechanisms have practically no effect on the pH of the desalted solution.

Thus, the CVC shape and the ability of ASE and CJMA-3 membranes to acidify the desalted solution are similar to the results we obtained earlier for other anion-exchange membranes (AMX, AMX-sb and AX by Astom, Japan) [51,52,54], which mainly contain quaternary ammonium fixed groups.

## References


1. Nefedova, G.Z.; Klimova, Z.G.; Sapoznikova, G.S. Ion-Exchange Membranes, Granulates, Powders. *Catalogue* **1977**.
2. Berezina, N.P.; Kononenko, N.A.; Dyomina, O.A.; Gnusin, N.P. Characterization of ion-exchange membrane materials: Properties vs structure. *Adv. Colloid Interface Sci.* **2008**, *139*, 3–28, doi:10.1016/j.cis.2008.01.002.
3. Simons, R. Electric field effects on proton transfer between ionizable groups and water in ion exchange membranes. *Electrochim. Acta* **1984**, *29*, 151–158, doi:10.1016/0013-4686(84)87040-1.
4. Simons, R. Water splitting in ion exchange membranes. *Electrochim. Acta* **1985**, *30*, 275–282, doi:10.1016/0013-4686(85)80184-5.
5. Zabolotskii, V.I.; Shel'deshov, N. V; Gnusin, N.P. Dissociation of Water Molecules in Systems with Ion-exchange Membranes. *Russ. Chem. Rev.* **1988**, *57*, 801–808, doi:10.1070/RC1988v057n08ABEH003389.
6. Lide, D.R.; Baysinger, G.; Berger, L.I.; Kehiaian, H. V; Roth, D.L.; Zwillinger, D.; Goldberg, R.N.; Haynes, W.M. *CRC Handbook of Chemistry and Physics*; CRC Press: New York, 1997;
7. Belashova, E.D.; Melnik, N.A.; Pismenskaya, N.D.; Shevtsova, K.A.; Nebavsky, A.V.; Lebedev, K.A.; Nikonenko, V.V. Overlimiting mass transfer through cation-exchange membranes modified by Nafion film and carbon nanotubes. *Electrochim. Acta* **2012**, *59*, 412–423, doi:10.1016/j.electacta.2011.10.077.
8. Helfferich, F.G. *Ion Exchange*; McGraw-Hill, 1962;

9. Lteif, R.; Dammak, L.; Larchet, C.; Auclair, B. Conductivitéélectrique membranaire: étude de l'effet de la concentration, de la nature de l'électrolyte et de la structure membranaire. *Eur. Polym. J.* **1999**, *35*, 1187–1195, doi:10.1016/S0014-3057(98)00213-4.
10. Kozmai, A.; Porozhnyy, M.; Ruleva, V.; Gorobchenko, A.; Pismenskaya, N.; Nikonenko, V. Is It Possible to Prepare a "Super" Anion-Exchange Membrane by a Polypyrrole-Based Modification? *Membranes (Basel).* **2023**, *13*, 103, doi:10.3390/membranes13010103.
11. Porozhnyy, M.; Huguet, P.; Cretin, M.; Safronova, E.; Nikonenko, V. Mathematical modeling of transport properties of proton-exchange membranes containing immobilized nanoparticles. *Int. J. Hydrogen Energy* **2016**, *41*, 15605–15614, doi:10.1016/j.ijhydene.2016.06.057.
12. Pismenskaya, N.D.; Nevakshenova, E.E.; Nikonenko, V. V. Using a Single Set of Structural and Kinetic Parameters of the Microheterogeneous Model to Describe the Sorption and Kinetic Properties of Ion-Exchange Membranes. *Pet. Chem.* **2018**, *58*, 465–473, doi:10.1134/S0965544118060087.
13. Zabolotsky, V.I.; Nikonenko, V.V. Effect of structural membrane inhomogeneity on transport properties. *J. Memb. Sci.* **1993**, *79*, 181–198, doi:10.1016/0376-7388(93)85115-D.
14. Titorova, V.; Sabbatovskiy, K.; Sarapulova, V.; Kirichenko, E.; Sobolev, V.; Kirichenko, K. Characterization of MK-40 Membrane Modified by Layers of Cation Exchange and Anion Exchange Polyelectrolytes. *Membranes (Basel).* **2020**, *10*, 20, doi:10.3390/membranes10020020.
15. Belova, E.I.; Lopatkova, G.Y.; Pismenskaya, N.D.; Nikonenko, V. V.; Larchet, C.; Pourcelly, G. Effect of Anion-exchange Membrane Surface Properties on Mechanisms of Overlimiting Mass Transfer. *J. Phys. Chem. B* **2006**, *110*, 13458–13469, doi:10.1021/jp062433f.
16. Ślęzak, A.; Bryll, A.; Grzegorczyn, S. A Numerical Study of the Hydrodynamic Stable Concentration Boundary Layers in a Membrane System Under Microgravitational Conditions. *J. Biol. Phys.* **2006**, *32*, 553–562, doi:10.1007/s10867-007-9037-0.
17. Moya, A.A.; Moleón, J.A. Study of the electrical properties of bi-layer ion-exchange membrane systems. *J. Electroanal. Chem.* **2010**, *647*, 53–59, doi:10.1016/j.jelechem.2010.05.011.
18. Moya, A.A. Electrochemical Impedance of Ion-Exchange Membranes in Ternary Solutions with Two Counterions. *J. Phys. Chem. C* **2014**, *118*, 2539–2553, doi:10.1021/jp4108238.
19. Rubinstein, I.; Zaltzman, B.; Futerman, A.; Gitis, V.; Nikonenko, V. Reexamination of

electrodiffusion time scales. *Phys. Rev. E* **2009**, *79*, 021506, doi:10.1103/PhysRevE.79.021506.

20. Nikonenko, V. V.; Kozmai, A.E. Electrical equivalent circuit of an ion-exchange membrane system. *Electrochim. Acta* **2011**, *56*, 1262–1269, doi:10.1016/j.electacta.2010.10.094.
21. Vorotyntsev, M.A.; Badiali, J.-P.; Inzelt, G. Electrochemical impedance spectroscopy of thin films with two mobile charge carriers: effects of the interfacial charging. *J. Electroanal. Chem.* **1999**, *472*, 7–19, doi:10.1016/S0022-0728(99)00253-3.
22. Vorotyntsev, M.A. Impedance of thin films with two mobile charge carriers. Interfacial exchange of both species with adjacent media. Effect of the double layer charges. *Electrochim. Acta* **2002**, *47*, 2071–2079, doi:10.1016/S0013-4686(02)00076-2.
23. *Impedance Spectroscopy*; Barsoukov, E., Macdonald, J.R., Eds.; Wiley, 2005; ISBN 9780471647492.
24. Sistat, P.; Kozmai, A.; Pismenskaya, N.; Larchet, C.; Pourcelly, G.; Nikonenko, V. Low-frequency impedance of an ion-exchange membrane system. *Electrochim. Acta* **2008**, *53*, 6380–6390, doi:10.1016/j.electacta.2008.04.041.
25. Muralidharan, V.S. Warburg impedance - basics revisited. *Anti-Corrosion Methods Mater.* **1997**, *44*, 26–29, doi:10.1108/00035599710157387.
26. Femmer, R.; Martí-Calatayud, M.C.; Wessling, M. Mechanistic modeling of the dielectric impedance of layered membrane architectures. *J. Memb. Sci.* **2016**, *520*, 29–36, doi:10.1016/j.memsci.2016.07.055.
27. Moya, A.A. Harmonic analysis in ideal ion-exchange membrane systems. *Electrochim. Acta* **2013**, *90*, 1–11, doi:10.1016/j.electacta.2012.12.031.
28. Kniaginicheva, E.; Pismenskaya, N.; Melnikov, S.; Belashova, E.; Sistat, P.; Cretin, M.; Nikonenko, V. Water splitting at an anion-exchange membrane as studied by impedance spectroscopy. *J. Memb. Sci.* **2015**, *496*, 78–83, doi:10.1016/j.memsci.2015.07.050.
29. Lévêque M. A. The laws of heat transmission by convection. *Les Ann. des Mines Mem.* **1928**, *12*, 201–299.
30. Newman J., T.-A.K.E. *Electrochemical systems*; John Wiley & Sons, 2012;
31. N.P. Gnusin, V.I. Zabolotskii, V.V. Nikonenko, M.K.U. Convective-Diffusion Model of Electrodialytic Desalination. Limiting Current and Diffusion Layer. *Sov. Electrochem.* **1986**, *23*, 273–278.
32. La Cerva, M.; Gurreri, L.; Tedesco, M.; Cipollina, A.; Ciofalo, M.; Tamburini, A.; Micale, G. Determination of limiting current density and current efficiency in electrodialysis units. *Desalination* **2018**, *445*, 138–148, doi:10.1016/j.desal.2018.07.028.

33. Nikonenko, V.; Nebavsky, A.; Mareev, S.; Kovalenko, A.; Urtenov, M.; Pourcelly, G. Modelling of Ion Transport in Electromembrane Systems: Impacts of Membrane Bulk and Surface Heterogeneity. *Appl. Sci.* **2018**, *9*, 25, doi:10.3390/app9010025.
34. Belashova, E.D.; Pismenskaya, N.D.; Nikonenko, V.V.; Sistat, P.; Pourcelly, G. Current-voltage characteristic of anion-exchange membrane in monosodium phosphate solution. Modelling and experiment. *J. Memb. Sci.* **2017**, *542*, 177–185, doi:10.1016/j.memsci.2017.08.002.
35. Urtenov, M.A.K.; Kirillova, E. V.; Seidova, N.M.; Nikonenko, V. V. Decoupling of the Nernst-Planck and Poisson equations. Application to a membrane system at overlimiting currents. *J. Phys. Chem. B* **2007**, doi:10.1021/jp073103d.
36. Kharkats, Y.I.; Sokirko, A.V. Theory of the effect of migration current exaltation taking into account dissociation-recombination reactions. *J. Electroanal. Chem.* **1991**, *303*, 27–44, doi:10.1016/0022-0728(91)85113-4.
37. Titorova, V.D.; Mareev, S.A.; Gorobchenko, A.D.; Gil, V.V.; Nikonenko, V.V.; Sabbatovskii, K.G.; Pismenskaya, N.D. Effect of current-induced coion transfer on the shape of chronopotentiograms of cation-exchange membranes. *J. Memb. Sci.* **2021**, *624*, 119036, doi:10.1016/j.memsci.2020.119036.
38. Lide, D.R. *CRC Handbook of Chemistry and Physics 86TH Edition 2005-2006*; 2005; ISBN 0849304792.
39. Wang, Y.; Zhang, Z.; Jiang, C.; Xu, T. Recovery of gamma-aminobutyric acid (GABA) from reaction mixtures containing salt by electrodialysis. *Sep. Purif. Technol.* **2016**, *170*, 353–359, doi:10.1016/j.seppur.2016.07.002.
40. Yan, H.; Wang, Y.; Xu, T. Developing Ion Exchange Membrane for Treating High Salinity Water Using Electrodialysis, Tianjin, China, 2019.
41. Sarapulova, V.; Pismenskaya, N.; Titorova, V.; Sharafan, M.; Wang, Y.; Xu, T.; Zhang, Y.; Nikonenko, V. Transport Characteristics of CJMAED$^{TM}$ Homogeneous Anion Exchange Membranes in Sodium Chloride and Sodium Sulfate Solutions. *Int. J. Mol. Sci.* **2021**, *22*, 1415, doi:10.3390/ijms22031415.
42. Ponomar, M.; Krasnyuk, E.; Butylskii, D.; Nikonenko, V.; Wang, Y.; Jiang, C.; Xu, T.; Pismenskaya, N. Sessile Drop Method: Critical Analysis and Optimization for Measuring the Contact Angle of an Ion-Exchange Membrane Surface. *Membranes (Basel).* **2022**, *12*, 765, doi:10.3390/membranes12080765.
43. Astom Detailed Specification of IEMs Produced Astom Corporation Available online: http://www.astom-corp.jp/en/product/10.html.
44. Chen, G.Q.; Wei, K.; Hassanvand, A.; Freeman, B.D.; Kentish, S.E. Single and binary ion

sorption equilibria of monovalent and divalent ions in commercial ion exchange membranes. *Water Res.* **2020**, *175*, 115681, doi:10.1016/j.watres.2020.115681.

45. Sugimoto, Y.; Ujike, R.; Higa, M.; Kakihana, Y.; Higa, M. Power Generation Performance of Reverse Electrodialysis (RED) Using Various Ion Exchange Membranes and Power Output Prediction for a Large RED Stack. *Membranes (Basel).* **2022**, *12*, 1141, doi:10.3390/membranes12111141.
46. Hernández-Pérez, L.; Martí-Calatayud, M.; Montañés, M.; Pérez-Herranz, V. Interplay between Forced Convection and Electroconvection during the Overlimiting Ion Transport through Anion-Exchange Membranes: A Fourier Transform Analysis of Membrane Voltage Drops. *Membranes (Basel).* **2023**, *13*, 363, doi:10.3390/membranes13030363.
47. Belloň, T.; Slouka, Z. Overlimiting behavior of surface-modified heterogeneous anion-exchange membranes. *J. Memb. Sci.* **2020**, *610*, 118291, doi:10.1016/j.memsci.2020.118291.
48. Zabolotskiy, V.I.; But, A.Y.; Vasil'eva, V.I.; Akberova, E.M.; Melnikov, S.S. Ion transport and electrochemical stability of strongly basic anion-exchange membranes under high current electrodialysis conditions. *J. Memb. Sci.* **2017**, *526*, 60–72, doi:10.1016/j.memsci.2016.12.028.
49. Simons, R. Electric field effects on proton transfer between ionizable groups and water in ion exchange membranes. *Electrochim. Acta* **1984**, *29*, 151–158, doi:10.1016/0013-4686(84)87040-1.
50. Pasechnaya, E.; Tsygurina, K.; Ponomar, M.; Chuprynina, D.; Nikonenko, V.; Pismenskaya, N. Comparison of the Electrodialysis Performance in Tartrate Stabilization of a Red Wine Using Aliphatic and Aromatic Commercial and Modified Ion-Exchange Membranes. *Membranes (Basel).* **2023**, *13*, 84, doi:10.3390/membranes13010084.
51. Pismenskaya, N.D.; Rybalkina, O.A.; Kozmai, A.E.; Tsygurina, K.A.; Melnikova, E.D.; Nikonenko, V.V. Generation of H+ and OH− ions in anion-exchange membrane/ampholyte-containing solution systems: A study using electrochemical impedance spectroscopy. *J. Memb. Sci.* **2020**, *601*, 117920, doi:10.1016/j.memsci.2020.117920.
52. Rybalkina, O.A.; Sharafan, M.V.; Nikonenko, V.V.; Pismenskaya, N.D. Two mechanisms of H+/OH− ion generation in anion-exchange membrane systems with polybasic acid salt solutions. *J. Memb. Sci.* **2022**, *651*, 120449, doi:10.1016/j.memsci.2022.120449.
53. Helfferich, F.G. Ion Exchange. *McGraw-Hill, New York,* **1962**.
54. Pismenskaya, N.; Rybalkina, O.; Moroz, I.; Mareev, S.; Nikonenko, V. Influence of Electroconvection on Chronopotentiograms of an Anion-Exchange Membrane in

Solutions of Weak Polybasic Acid Salts. *Int. J. Mol. Sci.* **2021**, *22*, 13518, doi:10.3390/ijms222413518.
